# Adeno-associated virus gene therapy for hemophilia: an update meta-analysis and systematic review

**DOI:** 10.3389/fmed.2025.1580264

**Published:** 2025-05-20

**Authors:** Wuxia Yang, Aidi Wang, Baoshan Liu

**Affiliations:** Department of Traditional Chinese Medicine, Tianjin Medical University General Hospital, Tianjin, China

**Keywords:** adeno-associated virus, gene therapy, hemophilia, systematic review meta-analysis, AAV

## Abstract

**Introduction:**

Remarkable clinical benefits have been achieved for patients with haemophilia through intravenously administered adeno-associated virus (AAV)-based liver-directed gene therapy. However, no comprehensive meta-analysis based on hemophilia types, various transgene types, AAV capsid, neutralizing antibody titers, and baseline factor VIII activity has been conducted to assess the efficacy of AAV vector gene therapy in hemophilic patients. We aimed to perform a systematic review and meta-analysis of the literature to assess the efficacy and safety of AAV-based gene therapy for hemophilia.

**Methods:**

We systematically searched PubMed, Clinical Trials, Medline, Web of Science, Embase, Cochrane Central Register of Controlled Trials, and Cochrane Database of Systematic Reviews for clinical trials involving patients diagnosed with hemophilia and treated with AAV gene therapy. The outcomes included annualized bleeding rate (ABR), annualized infusion rate (AIR), the incidence of treatment-related adverse events (TRAEs), alanine aminotransferase (ALT) elevation, and aspartate transaminase (AST) elevation.

**Results:**

A total of 13 articles were selected from 879 articles for meta-analysis. Pooled analyses showed that after gene therapy, the ABR was 1.10 and the AIR was 3.92, respectively. At 1 year after AAV gene therapy, all participants exhibited factor activity levels above their baseline values, with the highest level reaching 93.47%. Additionally, 50% of the hemophilia patients had elevated TRAEs, 50% had elevated ALT levels, and 29% had elevated AST levels. We also performed a subgroup analysis of these results according to different haemophilia types, various transgene types, AAV capsids, neutralizing antibody titers, and baseline factor VIII activity.

**Discussion:**

Our analysis supported the efficacy and safety of AAV-mediated gene therapy for hemophilia, providing a reference for clinical practice and the development of more gene therapy drugs.

## 1 Introduction

Hemophilia is an X-linked recessive bleeding disorder that results from a defect in the gene encoding coagulation factor (1). Its main feature is spontaneous bleeding, which, over repeated incidences, can lead to arthropathy, joint disability, or false tumors. These complications have a serious impact on patients’ quality of life and can even be life-threatening (2). Hemophilia A and B represent the most common and widely recognized types of hemophilia. Hemophilia A occurs in 1 in 5,000 live male births, making it approximately six times more common than hemophilia B (3). The severity classification of the disease is dependent on the level of factor IX activity: individuals with <1% factor IX activity are classified as severe, those with 1–5% as moderately severe, and those with 5–40% as having mild hemophilia. In its most severe form (circulating factor IX activity levels of <1%), symptoms can become apparent early in life (4). The ultimate dream for anyone with a serious disease is to cure the disease, and hemophilia is no exception. In contrast to the conventional frequent coagulation factor infusion treatment, gene therapy replaces or corrects damaged genes in target cells by introducing genetic material into those cells using viral or non-viral vectors. Consequently, it is the first treatment that can provide a stable, sustained, long-term factor level of over 15% and even more than 40% in some patients (5). At present, adeno-associated virus (AAV) has been extensively studied as gene therapy vectors (6). Three AAV-based gene therapy drugs, including Roctavian, Hemgenix, and BeQvez, have been approved for the treatment of adult hemophilia. Many trials have reported increased factor activity, reduced bleeding episodes, and decreased dependency on factor replacement following AAV-mediated factor gene therapy. Therefore, a growing number of clinical trials utilizing AAV vectors for *in vivo* gene therapy are currently underway.

However, limited sample sizes, non-randomized designs, varied doses, AAV capsids, and transgene may have contributed to assessment bias and a lack of knowledge on the safety and effectiveness of AAV-based gene therapy for hemophilia. As of yet, no comprehensive meta-analysis based on various transgene types, AAV capsid, neutralizing antibody titers, and baseline factor VIII activity has been conducted to assess the efficacy and safety of AAV vector gene therapy in hemophilic patients. By conducting a comprehensive review and meta-analysis of existing studies, we integrated safety and efficacy data on AAV-based hemophilia gene therapy to fill this research gap. Significant evidence from our analysis supports the development and clinical application of AAV-based gene therapy for the treatment of hemophilia.

## 2 Methods

### 2.1 Search strategy and selection criteria

We included studies that reported data on the outcomes of hemophilia A or hemophilia B patients who were treated with AAV gene therapy. The most recently updated results of each trial were analyzed, either from published articles or from conference proceedings. First, we searched PubMed, Clinical Trials, Medline, Web of Science, Embase, Cochrane Central Register of Controlled Trials, and Cochrane Database of Systematic Reviews before July 2024, using the search terms “AAV,” “adeno-associated virus,” “gene therapy,” “hemophilia,” “hemophilia A,” and “hemophilia B.” Second, we assessed the articles’ reference lists and applied a rigorous selection procedure to find the most relevant research. In addition, we hand-searched abstracts from relevant conference proceedings (American Society of Hematology, American Society of Clinical Oncology, European Hematology Association, European Society of Medical Oncology, American Society for Transplantation and Cellular Therapy, European Society for Blood and Marrow Transplantation, and American Association for Cancer Research). Moreover, we discarded trials that were published in languages other than English, had duplicate data, did not have outcomes that were fully complete after AAV-based gene therapy or were irrelevant. Animal experimental data were also excluded. Finally, two reviewers managed and analyzed all studies using Zotero independently. The search was performed in accordance with the Preferred Reporting Items for Systematic Reviews and Meta-Analyses and registered with the International Prospective Register of Systematic Reviews (PROSPERO registration ID: CRD42024572094).

### 2.2 Data extraction

These data were extracted independently by one reviewer using a predesigned data extraction form. In the process of data analysis, the following data were extracted: characteristics of the trial (authors, title, and year); baseline characteristics of patients and diseases (sample size, factor VIII activity, neutralizing antibody titers, annualized bleeding rate, annualized number of FVIII infusions); intervention doses. Data extraction differences were resolved either by consensus or by a third party.

### 2.3 Quality assessment

The quality of all studies was independently evaluated by two researchers using the methodological index for non-randomized studies (MINORS) (7). The MINORS instrument is made up of 12 items in total. The first eight items are only related to non-comparative studies and are used to evaluate the methodological quality of non-randomized clinical research. These eight items are evaluated using a scoring system (0, not reported;1, reported but inadequate; or 2, reported and adequate), with an ideal score of 16 for non-comparative studies. Disagreements were resolved through discussion by a third researcher.

### 2.4 Statistical analysis

All data in this meta-analysis were analyzed with STATA 14.2 software. We estimated from each study cohort the cumulative effect size (event rate) and 95% CI for each outcome. We pooled event rates for each intervention in a meta-analysis using a random-effects model to incorporate heterogeneity in the analysis (8) and the Freeman-Tukey double arcsine transformation to stabilize the variance of proportions (9). We assess the heterogeneity with I^2^ statistics. I^2^ values were defined as low heterogeneity (>25%, up to 50%), moderate heterogeneity (>50%, up to 75%), or high heterogeneity (>75%) (10). Prespecified subgroup analyses were done for different hemophilia types including hemophilia A and hemophilia B, AAV capsid, transgene, baseline factor VIII activity, and neutralizing antibody titers. We used the Cochran’s Q-test to evaluate the heterogeneity between subgroups. Q was calculated as the weighted sum of squared differences between each subgroup and the pooled effect across subgroups, with the weights being those used in the pooling method.

## 3 Results

### 3.1 Results of the database search

**Figure 1:**
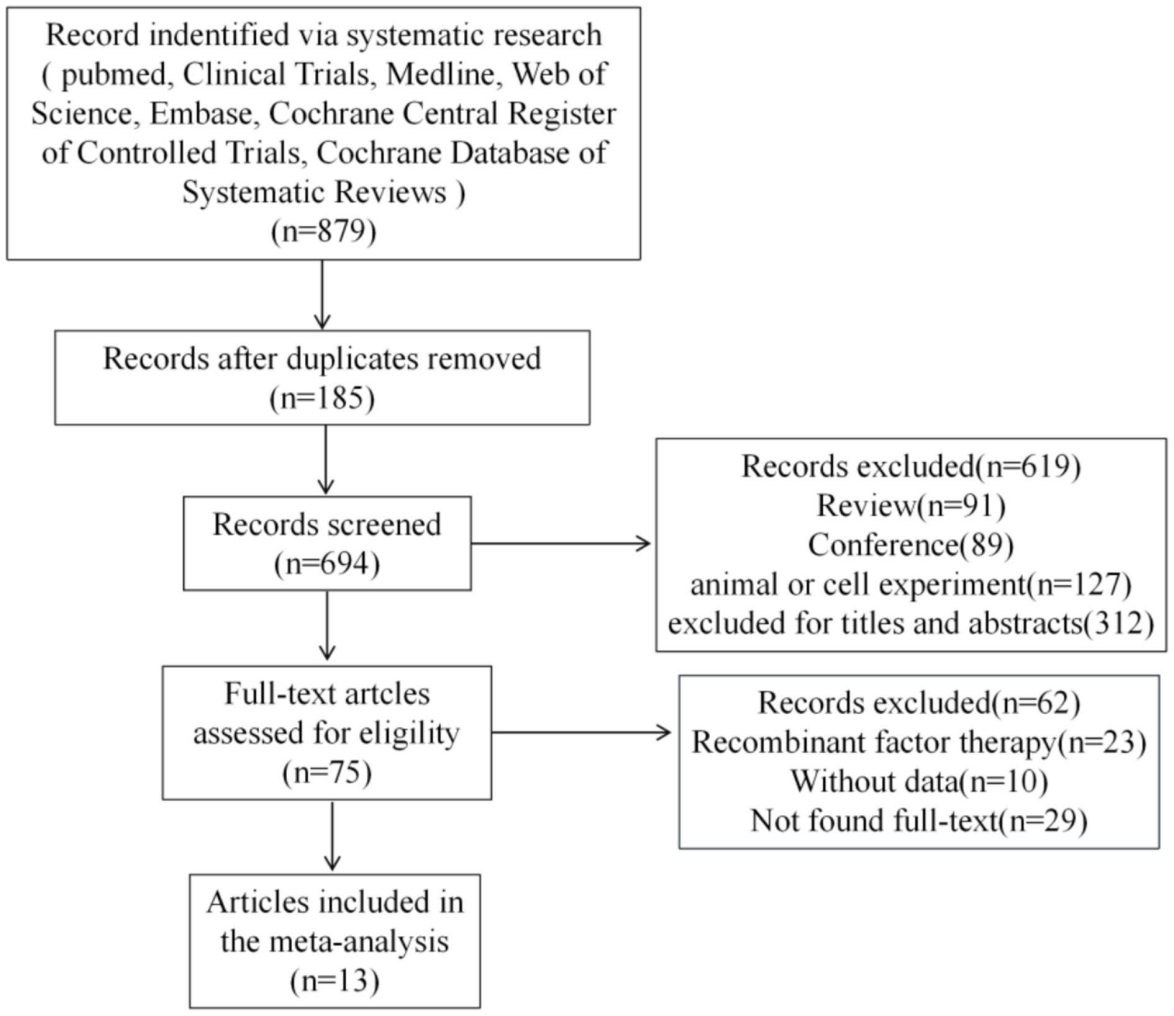
Flow diagram of study scan and selection process.

Figure 1 shows the scan flowchart for academic rescan. The scan strategy detected 879 potentially relevant citations. Meticulous screening excluded 694 studies due to duplication. Of the remaining studies, 619 were excluded because they did not meet the inclusion criteria, such as inadequate outcome reporting, unable to link to clinical trials, and so on. An additional 62 references were excluded after a detailed evaluation of the entire text. Finally, 13 studies satisfied the eligibility criteria and were included in the systematic review and meta-analysis (11–23). All included studies achieved MINORS scores greater than 12, indicating a satisfactory level of studies quality. Detailed results are presented in Supplementary material 7.

### 3.2 Characteristics of the included study selection

An overview of the features involved in the 13 trials is given in Table 1, with 6 trials for hemophilia A and 7 for hemophilia B. These single-arm studies, which encompassed a total of 410 patients, used a variety of more than 5 diverse vector types, and 4 transgenes, and were administered over 10 distinct dosages of AAV-based therapies.

**Table 1:** Overall characteristics of the 13 trials included in the analysis.

Name	Year	Study registration number	AAV capsid	Transgene	Baseline factor VIII activity	Neutralizing antibody titers	Dose	*N*	Outcomes	Factor VIII activity level 1 year
Hemophilia A										
Savita RJ	2017	NCT02576795	AAV5-hFVIII-SQ	Codon-optimized BDD-F VII	≤1	Negative	6 × 10^13^vg/kg	7	①②③④⑤⑥	93 ± 48
Pasi KJ	2020	NCT02576795	AAV5-hFVIII-SQ	Codon-optimized BDD-F VII	≤1	Negative	4 × 10^13^vg/kg	6	①②③④	20.83 + 11.38
M.C. Ozelo, J	2022	NCT03370913	AAV5-hFVIII-SQ	Codon-optimized BDD-F VII	<1	Negative	6 × 10^13^vg/kg	132	①②③④⑤⑥	CS:42.9 ± 45.5
Mahlangu J	2023	NCT03370913	AAV5-hFVIII-SQ	Codon-optimized BDD-F VII	<1	Negative	6 × 10^13^vg/kg	132	①④⑤⑥	NA
George LA	2021	NCT03003533 NCT03432520	AAV-Spark 200	BDD-F VII	≤2	≤1:5	5 × 10^11^vg/kg	2	②③④	OS:9.5 ± 1.5 CS:5.5 ± 0.5
							1 × 10^12^ vg/kg	3	②③④	OS:11.7 ± 17.2 CS:6.7 ± 1.8
							1.5 × 10^12^vg/kg	4	②③④⑤	OS:12 ± 4.5 CS:7.3 ± 2.2
							2 × 10^12^ vg/kg	9	②③④⑤	OS:9 ± 2.3 CS:5.4 ± 2.7
Andrew D	2024	NCT03061201	rAAV6	BDD-F VII	<1	Negative	3 × 10^13^vg/kg	5	①③④⑤⑥	CS: 42.6 (7.8–122.3)
Hemophilia B										
Nathwani AC	2011	NCT00979238	AAV8	Codon-optimized FIX	<1	Negative	2 × 10^11^vg/kg 6 × 10^11^vg/kg 2 × 10^12^ vg/kg	6	④⑤⑥	NA
Nathwani AC	2014	NCT00979238	AAV8	Codon-optimized FIX	<1	Negative	2 × 10^11^vg/kg 6 × 10^11^vg/kg 2 × 10^12^ vg/kg	10	②③④⑤	OS:3.91 ± 0.87
Chowdary P	2022	NCT03369444	AAVS3	Codon-optimized FIXcontaining the Padua mutation	<2	Negative	3.84 × 10^11^vg/kg 6.4 × 1011vg/kg 8.32 × 10^11^vg/kg 1.28 × 10^12^vg/kg	10	①③④⑤⑥	70.18 ± 68.66
George LA	2017	NCT02484092	AAV-Spark100	Codon-optimized FIX containing the Padua mutation	<2	≤1:5	5 × 10^11^vg/kg	10	①②③④⑤	33.7 ± 16.77
Miesbach W	2018	NCT02396342	AAV5	Codon-optimized FIX	≤1.5	Negative	5×10^12^ vg/kg	5	①②③④⑤	4.4 ± 1.86
							2 × 10^13^vg/kg	5	①②③④⑤	6.96 ± 2.88
Xue F	2022	NCT04135300	AAV843	Codon-optimized FIX containing the Padua mutation	<2	≤1:4	5 × 10^12^ vg/kg	10	①②③④⑤⑥	OS:36.93 ± 20.49
Pipe SW	2023	NCT03569891	AAV5	Codon-optimized FIX containing the Padua mutation	≤2	Maximum titer:3212.3	2 × 10^13^vg/kg	54	①②③④⑤⑥	OS:39 ± 18.7

FVIII activity data from one-stage clot (OS)assay, chromogenic-substrate (CS) assay, and one-stage aPTT, the text in the table that is not marked indicates that it was not mentioned in the original text; ABR: annualized bleeding rate; AIR: annualized infusion rate; TRAEs: the incidence of treatment-related adverse events; ABR and AIR: The contrast values before and after treatment are ABR and AIR.

NA, not available; N, number of patients included; Outcomes, ① ABR; ② AIR; ③ factor activity level 1 year; ④ treated-related adverse events; ⑤ ALT elevation; and ⑥ AST elevation.

### 3.3 Annualized bleeding rate (ABR) at 1 year after adeno-associated virus gene therapy

**Figure 2:**
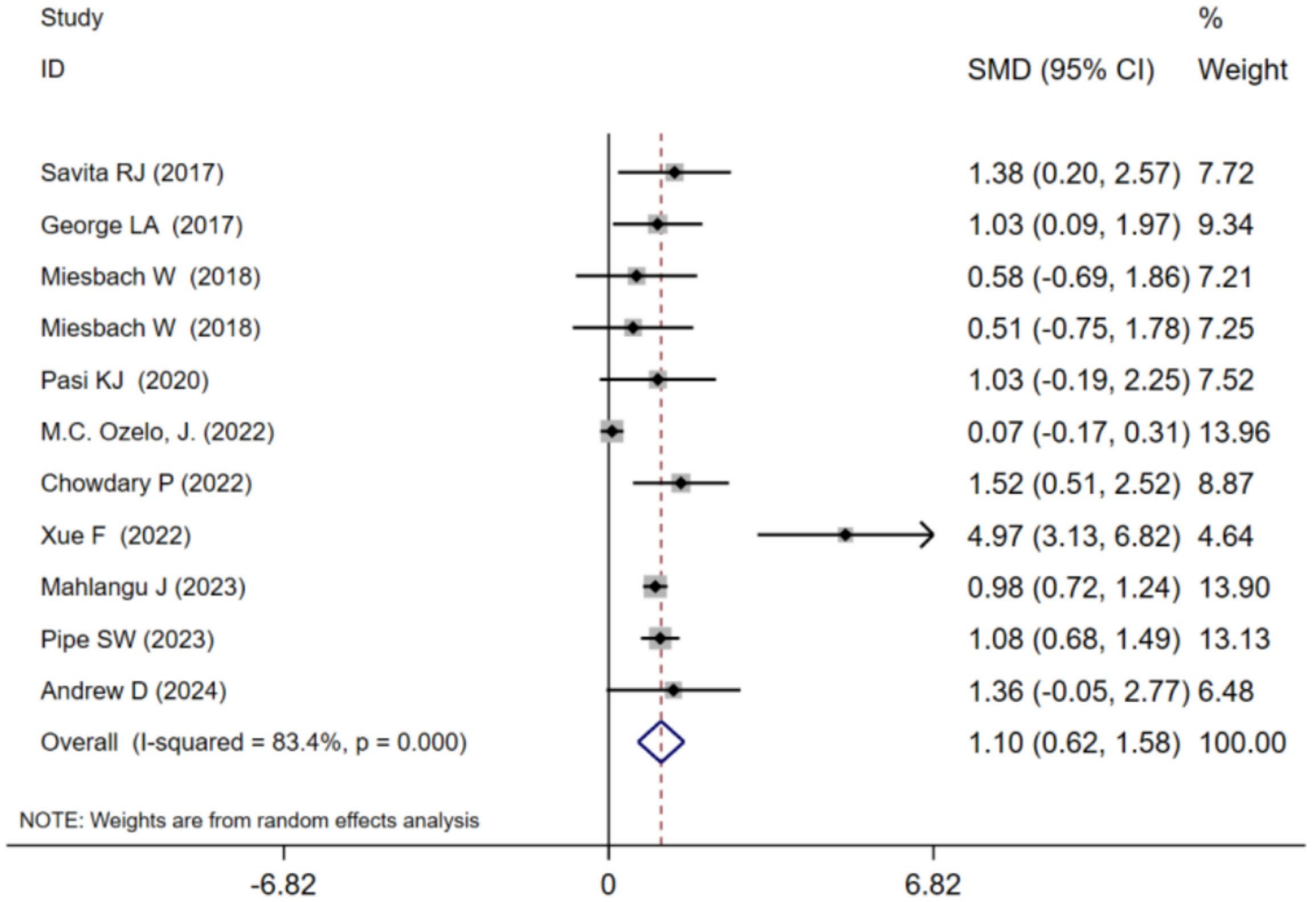
Overall ABR at 1 year after AAV gene therapy.

ABR at 1 year after adeno-associated virus gene therapy was observed in 11 studies. A random-effects model was chosen due to significant heterogeneity (*I*^2^ = 83.4%, *p* < 0.001). The results of the analysis indicated a pooled Standardized Mean Difference(SMD) of 1.10 (95% CI: 0.62–1.58)(Table 2; Figure 2). Further analysis examined the SMD concerning different types of hemophilia, AAV capsid, transgene, baseline factor VIII activity, and neutralizing antibody titers. Subgroup analysis 1 demonstrated that the pooled SMD in hemophilia A patients was 0.76 (95% CI: 0.06–1.45), while in hemophilia B patients, it was 1.40 (95% CI: 0.61–2.18) (Table 2; Supplementary Figure 2B). Subgroup analysis 2 demonstrated that the pooled SMD in the AAV5 capsid was 0.77 (95% CI: 0.28–1.26), while it was 2.04 (95% CI: 0.69–3.39) in other AAV capsids (Table 2; Supplementary Figure 2C). Subgroup analysis 3 demonstrated that the pooled SMD in codon-optimized BDD-FVII was 1.83 (95% CI: 0.75–2.91), while it was 0.76 (95% CI: 0.06–1.45) in codon-optimized FIX containing the Padua mutation (Table 2; Supplementary Figure 2D). Subgroup analysis 4 demonstrated that the pooled SMD in neutralizing antibody titers negative was 0.85 (95% CI: 0.34–1.36), while it was 2.06 (95% CI: 0.51–3.62) in neutralizing antibody titers positive (Table 2; Supplementary Figure 2E). Subgroup analysis 5 demonstrated that the pooled SMD in baseline factor VIII activity (<2/≤2) 1.83 (95% CI: 0.75–2.91), baseline A factor VIII activity (≤1.5/≤1) 0.90 (95% CI: 0.28–1.52), while it was 0.68 (95% CI: −0.12–1.47) in baseline factor VIII activity (<1) (Table 2; Supplementary Figure 2F).

**Table 2:** Results of subgroup analysis.

	ABR	AIR	TRAEs	ALT elevation	AST elevation
Total	1.10 (0.62, 1.58)	3.92 (2.87, 4.98)	0.47 (0.25, 0.69)	0.50 (0.27, 0.72)	0.29 (0.17, 0.41)
Hemophilia types					
Hemophilia A	0.76 (0.06, 1.45)	5.09 (2.51, 7.68)	0.657 (0.398, 0.879)	0.532 (0.251, 0.805)	0.402 (0.336, 0.470)
Hemophilia B	1.40 (0.61, 2.18)	3.76 (2.11, 5.41)	0.413 (0.159, 0.691)	0.336 (0.053, 0.72)	0.131 (0.056, 0.225)
AAV capsid					
AAV5	0.77 (0.28, 1.26)	4.78 (3.64, 5.93)	0.586 (0.323, 0.828)	0.482 (0.161, 0.810)	0.339 (0.188, 0.506)
Other	2.04 (0.69, 3.39)	2.38 (0.65, 4.11)	0.376 (0.129, 0.655)	0.399 (0.091, 0.754)	0.131 (0.056, 0.225)
Transgene					
Codon-optimized BDD-F VII	1.83 (0.75, 2.91)	5.19 (1.39, 9.00)	0.465 (0.173, 0.768)	0.524 (0.272, 0.771)	0.123 (0.050, 0.217)
Codon-optimized FIX containing the Padua mutation	0.76 (0.06, 1.45)	3.10 (0.75, 5.45)	0.418 (0.093, 0.784)	0.354 (0.005, 0.838)	0.406 (0.340, 0.473)
BDD-F VII	NA	5.09 (2.51, 7.68)	NA	NA	NA
Neutralizing antibody titers					
Negative	0.85 (0.34, 1.36)	4.66 (3.18, 6.14)	0.539 (0.276, 0.793)	0.710 (0.537, 0.859)	NA
Other	2.06 (0.51, 3.62)	3.10 (0.75, 5.45)	0.341 (0.076, 0.666)	0.179 (0.093, 0.283)	NA
Baseline factor VIII activity					
<2/≤2	1.83 (0.75, 2.91)	3.10 (0.75, 5.45)	0.428 (0.168, 0.708)	0.300 (0.004, 0.747)	0.123 (0.50, 0.217)
≤1.5/≤1	0.90 (0.28, 1.52)	6.50 (4.70, 8.30)	0.313 (0.056, 0.636)	NA	NA
<1	0.68 (−0.12, 1.47)	NA	0.855 (0.673, 0.979)	0.574 (0.332, 0.800)	0.395 (0.329, 0.)

ABR, annualized bleeding rate; AIR, annualized infusion rate; TRAEs, the incidence of treatment-related adverse events; ABR and AIR, The contrast values before and after treatment are ABR and AIR.

AAV, other include AAV-Spark 200; AAV-Spark100; rAAV6; AAV8; AAVS3; AAV843; Neutralizing antibody titers: other include≤1:5;≤1:4;maximum titer:3212.3; All of the subgroup analysis’s findings came from outcomes that were more than or equal to three.

NA, not available; N, number of patients included.

### 3.4 Annualized infusion rate(AIR) at 1 year after adeno-associated virus gene therapy

**Figure 3:**
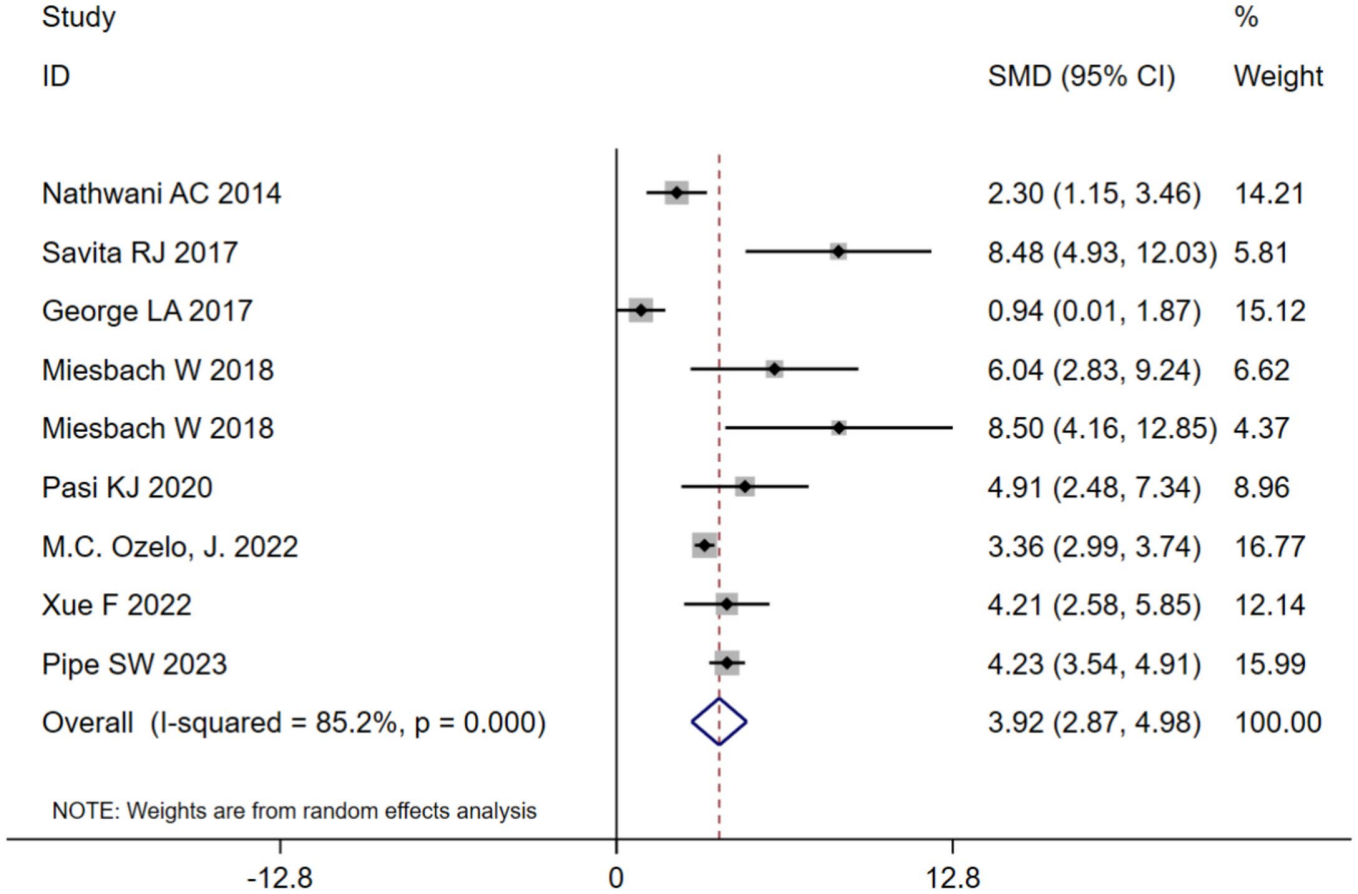
Overall AIR at 1 year after AAV gene therapy.

AIR at 1 year after adeno-associated virus gene therapy was observed in 9 studies. A random-effects model was chosen due to significant heterogeneity (*I*^2^ = 85.2%, *p* < 0.001). The results of the analysis indicated a pooled SMD of 3.92 (95% CI: 2.87–4.98) (Table 2; Figure 3). Further analysis examined the SMD concerning different types of hemophilia, AAV capsid, transgene, baseline factor VIII activity, and neutralizing antibody titers. Subgroup analysis 1 demonstrated that the pooled SMD in hemophilia A patients was 5.09 (95% CI: 2.51–7.68), while it was 3.76 (95% CI: 2.11–5.41) in hemophilia B patients (Table 2; Supplementary Figure 3B). Subgroup analysis 2 demonstrated that the pooled SMD in the AAV5 capsid was 4.78 (95% CI: 3.64–5.93), while it was 2.38 (95% CI: 0.65–4.11) in other AAV capsids (Table 2; Supplementary Figure 3C). Subgroup analysis 3 demonstrated that the pooled SMD in codon-optimized BDD-FVII was 5.19 (95% CI: 1.39–9.00), BDD-FVII was 5.09 (95% CI: 2.51–7.68), while it was 3.10 (95% CI: 0.75–5.45) in codon-optimized FIX containing the Padua mutation (Table 2; Supplementary Figure 3D). Subgroup analysis 4 demonstrated that the pooled SMD in neutralizing antibody titers negative was 4.66 (95% CI: 3.18–6.14), while it was 3.10 (95% CI: 0.75–5.45) in neutralizing antibody titers positive (Table 2; Supplementary Figure 3E). Subgroup analysis 5 demonstrated that the pooled SMD in baseline factor VIII activity (<2/≤2) 3.10 (95% CI: 0.75–5.45), while it was 6.50 (95% CI: 4.70–8.30) baseline factor VIII activity (≤1.5/≤1) (Table 2; Supplementary Figure 3F).

### 3.5 Factor activity level at 1 year after AAV gene therapy

Over time, expectations for feasible factor levels have evolved for both physicians and patients. The initial aim was to stop spontaneous joint bleeding by reaching levels of 5 IU/dL. Endogenous factor activity during the 1 year after treatment is shown in Table 1. At diagnosis, all participants had factor IX activity of less than or equal to 2%, which 5 participants (26.3%) had factor IX activity of less than 1%. The results of the pooled studies indicate that factor activity levels at 1-year post-gene therapy range from a minimum of 3.91 ± 0.87 to a maximum of 93 ± 48. The comparison of factor activity using two distinct methods, the one-stage clot (OS) assay, and the chromogenic-substrate (CS) assay. Each method has its unique advantages and limitations, influencing the interpretation of factor activity levels (24). Furthermore, there is a significant association between the level of coagulation factor activity and the dose used, especially when the maximum dose is reached, which essentially follows the pattern of the dose–effect relationship, as shown in Table 2.

### 3.6 Safety of AAV therapy

**Figure 4:**
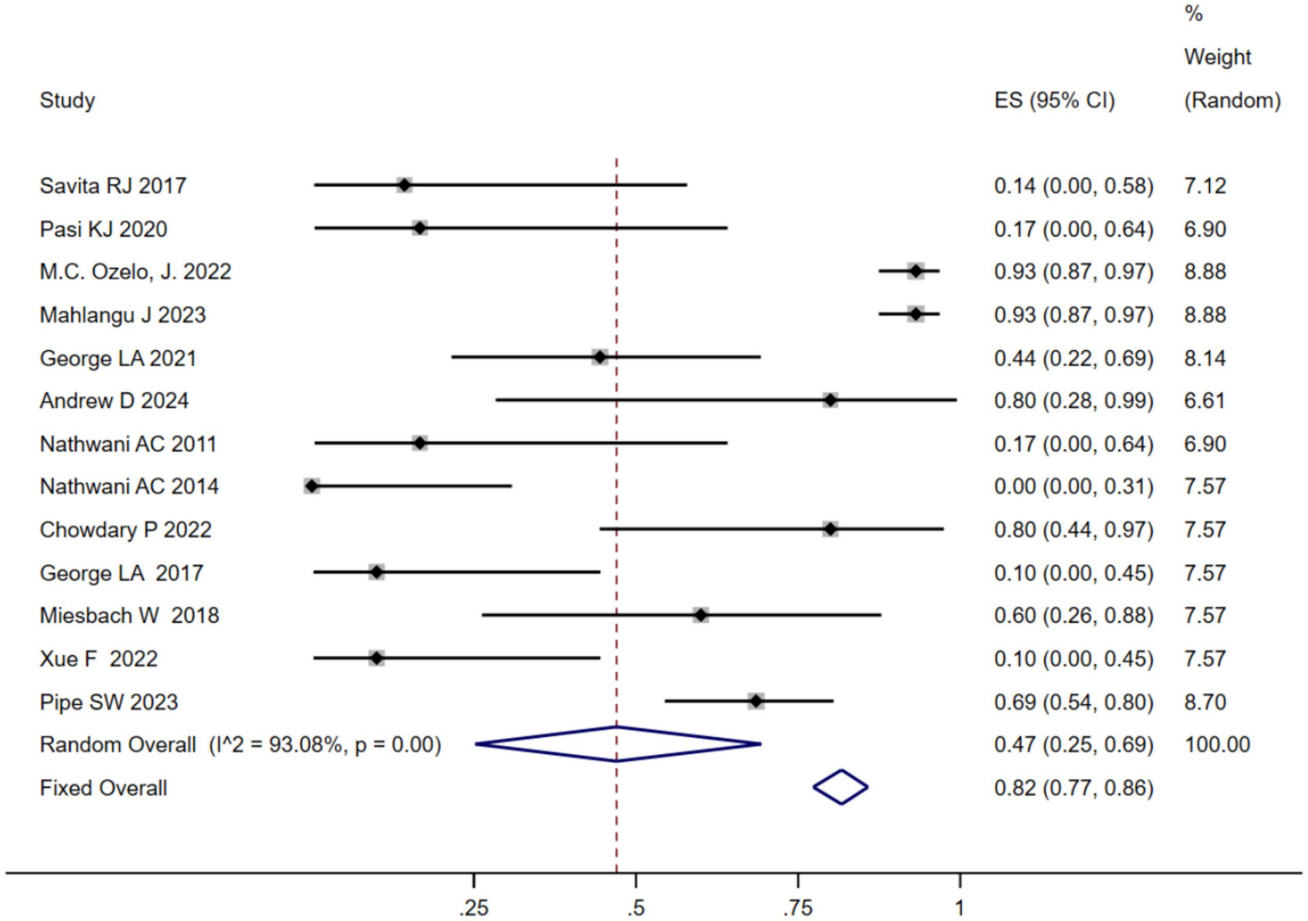
TRAEs associated with AAV gene therapy.

**Figure 5:**
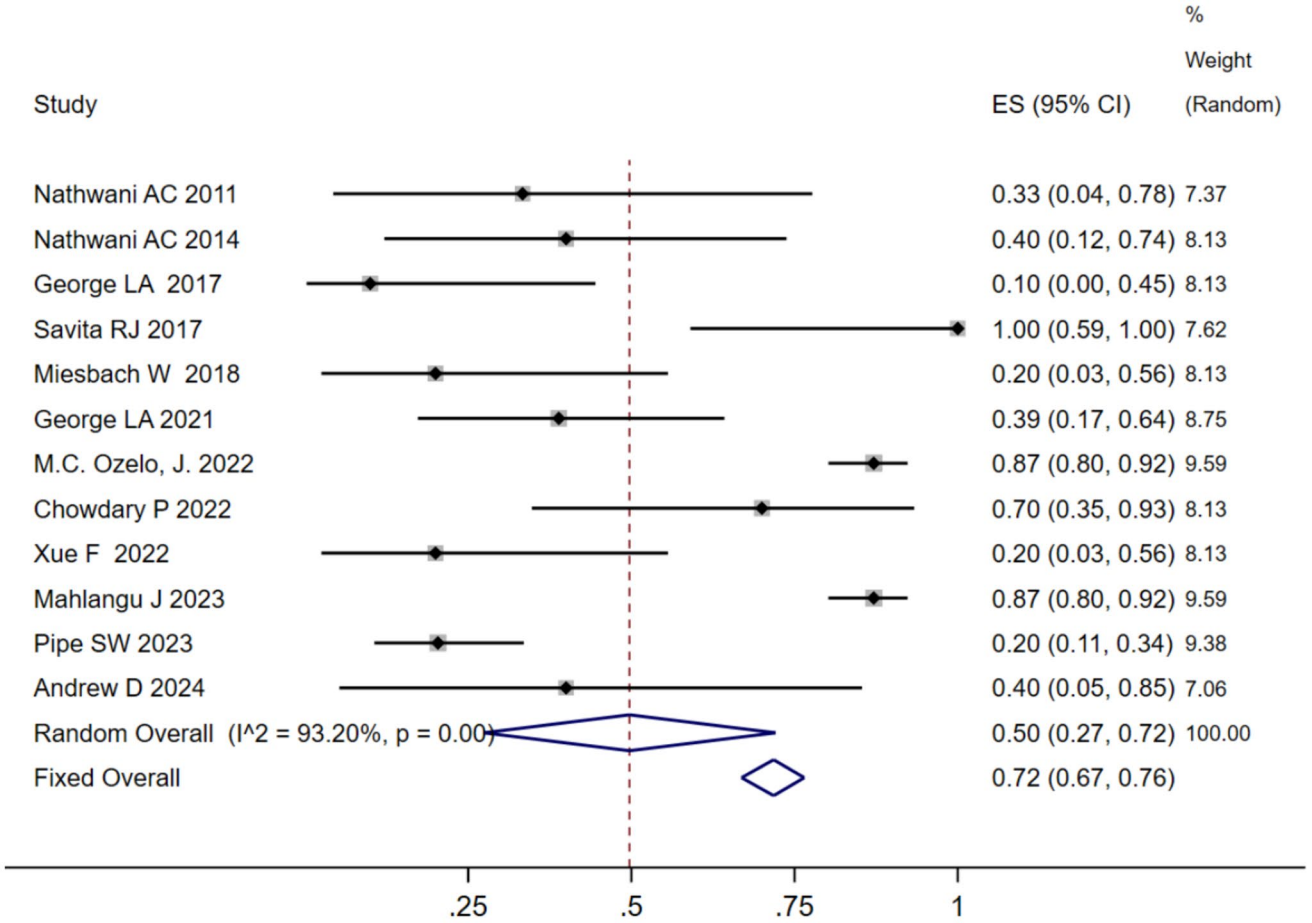
ALT elevation after AAV gene therapy.

**Figure 6:**
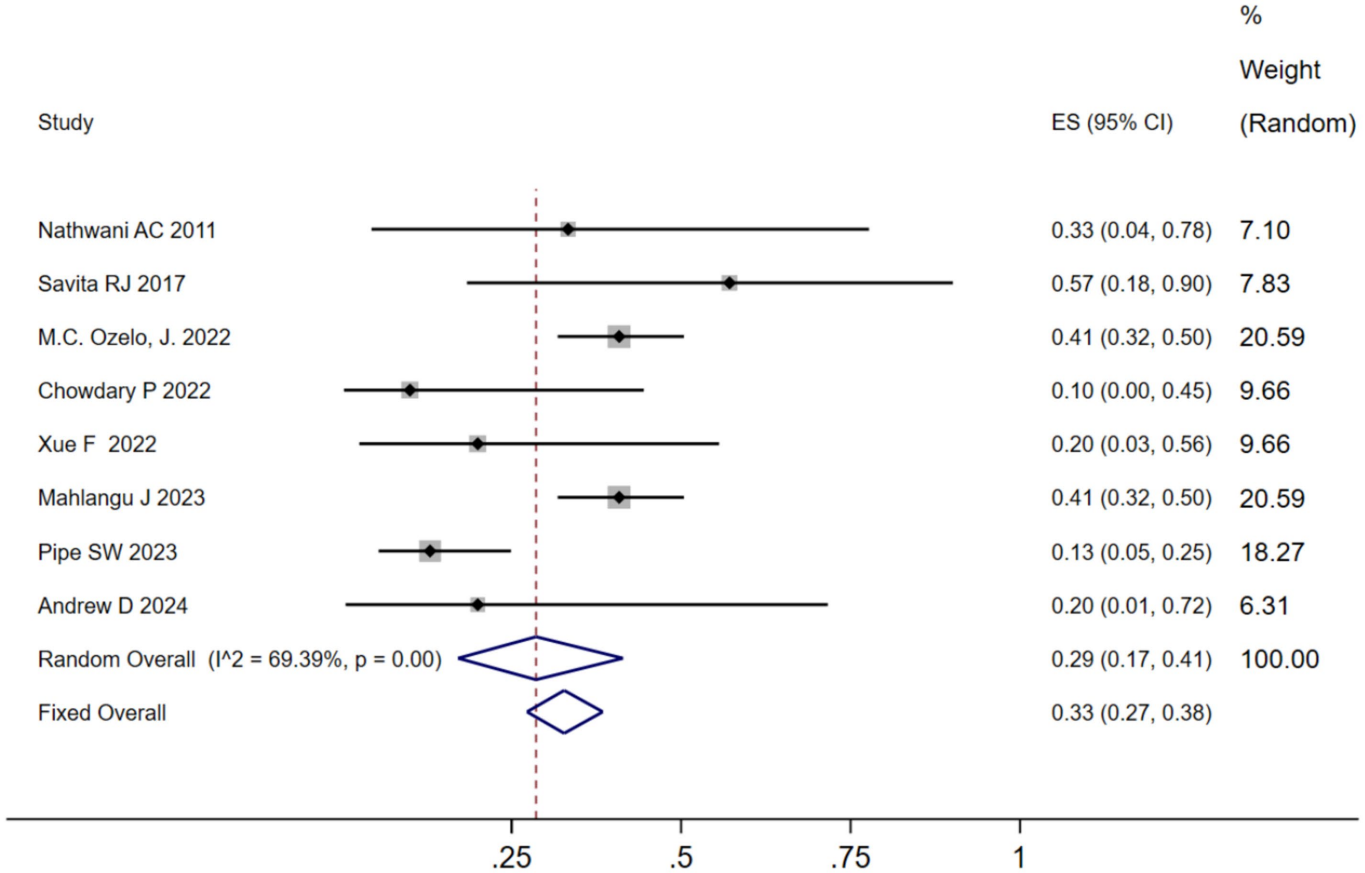
AST elevation after AAV gene therapy.

We analyzed the TRAEs associated with AAV-based gene therapy for hemophilia. The pooled data revealed the incidence of TRAEs as 0.47 (95% CI: 0.25, 0.69), with significant heterogeneity (*I*^2^ = 93.08%, *p* < 0.01) (Figure 4). In hemophilia A, the incidence of TRAEs was 0.657 (95% CI: 0.398–0.879), and in hemophilia B, the incidence of TRAEs was 0.413 (95% CI: 0.159–0.691) (Table 2; Supplementary Figure 4B). Subgroup analysis demonstrated that AAV5 capsid was 0.586(95% CI: 0.323–0.828), while it was 0.376(95% CI: 0.129–0.655) in other AAV capsids (Table 2; Supplementary Figure 4C). Subgroup analysis demonstrated that codon-optimized BDD-FVII was 0.456 (95% CI: 0.173–0.768), while it was 0.418 (95% CI: 0.093–0.784) in codon-optimized FIX containing the Padua mutation (Table 2; Supplementary Figure CD). Subgroup analysis demonstrated that neutralizing antibody titers negative was 0.539 (95% CI: 0.276–0.793), while it was 0.341(95% CI: 0.076–0.666) in neutralizing antibody titers positive (Table 2; Supplementary Figure 4E). Subgroup analysis demonstrated that baseline factor VIII activity (<2/≤2) 0.428 (95% CI: 0.168–0.708), baseline factor VIII activity (≤1.5/≤1) 0.313 (95% CI: 0.056–0.636), while it was 0.855 (95% CI: 0.673–0.979) in baseline factor VIII activity (<1) (Table 2; Supplementary Figure 4F). Further investigation of AEs showed that the incidence of ALT and AST elevation in the pooled data was 0.50 (95% CI: 0.27–0.72) and (95% CI: 0.17–0.41), respectively (Figures 5, 6). The results of the other subgroup analyses are presented in Table 2 and Supplementary material 5.

## 4 Discussion

The current standard of care is the recurrent infusion of FVIII or FIX concentrate to improve hemostasis, administered in response to bleeding or prophylactically to prevent hemorrhage. Practically, this requires intravenous infusions of FVIII or FIX concentrate several times weekly to maintain factor activity troughs above 1% of normal, though these great improvements with the advent of extended-half-life factor products (25, 26). Over the past few decades, remarkable breakthroughs and clinical benefits have been achieved in patients with hemophilia through intravenously administered AAV-based liver-directed gene therapy. The effects of gene therapy may be long-lasting without the need for repeated interventions (27). The AAV vectors are usually preferred for *in vivo* gene therapy due to several advantages, including the ability to transduce both dividing and quiescent cells, robust in vivo transduction efficiency, long-term transgene expression in quiescent cells, tropism for specific tissues and cell types, relatively low immunogenicity, non-pathogenicity, and a history of clinical safety (28). Nowadays, great clinical benefit has been achieved in patients with hemophilia A and B through intravenously administered AAV-based liver-directed gene therapy. Although some studies have previously reviewed AAV gene therapy for hemophilia (29–31), as of yet, no comprehensive meta-analysis based on various transgene types, AAV capsid, neutralizing antibody titers, and baseline factor VIII activity has been conducted to assess the efficacy of AAV vector gene therapy in hemophiliac patients. We aimed to gain a more exhaustive and accurate comprehension of the effect of this novel therapy in hemophilia management by taking advantage of more elaborate statistical analysis methods of the included studies and to provide data support for the development of gene therapy drugs for hemophilia. ABR and AIR are commonly utilized as endpoints to assess the efficacy of therapy in clinical trials involving individuals with hemophilia. According to our pooled data, AAV-based gene therapy resulted in reduced ABR and AIR among hemophilia patients. The subgroup analysis indicated that different hemophilia types, AAV capsid, transgene, neutralizing antibody titers, and baseline factor VIII activity led to slight differences in the results. The severity of bleeding in hemophilia patients is mainly related to the level of factor activity. In the studies included in our analysis, all participants were diagnosed with factor IX activity levels of less than or equal to 2%. Of these, 5 participants (26.3%) had factor IX activity levels below 1%. The results of the pooled studies indicate that factor activity levels are apparently elevated at 1-year post-gene therapy, despite the differences in the data from the different detection methods. As Ozelo et al. (13) suggested that AAV-modulated gene therapy enabled stable endogenous factor activity without requiring regular prophylactic treatment with factor infusions. Furthermore, the efficacy outcomes are significantly impacted by individual variations in the effects of gene therapy and by varying doses. Additionally, it is important to note that variability in the range of FIX activity has been reported between OS assays conducted with different reagents, as well as between OS and CS assays conducted at different testing sites (32). These discrepancies arise from differences in standardization, reagent sensitivity, and interference profiles (33), more standardized tests will be needed to assess the exact efficacy of gene therapy in the future. It is noteworthy that we conducted a combined analysis of the specific values in which AAV-based gene therapy altered ABR, AIR, and factor IX activity, and performed a subgroup analysis of them. However, the efficacy results should be interpreted with caution. This caution is due to several factors: the greater emphasis on outcome measures compared to other studies, the absence of comparable data, small sample size, differences in measurement methods, inadequate duration of long-term follow-up, preselection bias in the enrolled subjects, and various confounders that may have influenced the results.

Safety is a critical outcome of interest in clinical trials, with an incidence of 0.47 for TRAEs. Our comprehensive analysis found that nearly all studies reported increased ALT levels as an AE following gene therapy, and some studies have also reported elevated AST levels. Our meta-analysis revealed that the incidence of ALT and AST elevation resulting from AAV-based gene therapy in hemophilia patients was estimated at 0.50 and 0.29, respectively. The subgroup analysis indicated that different hemophilia types, AAV capsid, transgene, neutralizing antibody titers, and baseline factor VIII activity led to slight differences in the results. Several potential causes of elevated ALT and AST levels include the interaction of cytotoxic T lymphocytes with the AAV capsid, which induces an immune response in transduced hepatocytes (34). Additionally, the type and dose of vector used for gene delivery, release of cytokines during delivery, number of CpG motifs, and use of other potentially hepatotoxic drugs may also contribute to increased ALT and AST levels (27, 35). Despite the acceptable safety profile of AAV-mediated gene therapy in hemophilia patients being acceptable, it must be emphasized that the majority of studies conducted thus far have comprised small cohorts, which increases the potential for imprecision. Therefore, large-scale studies are necessary to gain further insight into the safety of AAV-based gene therapy for hemophilia patients.

Our meta-analysis, based on various transgene types, AAV capsid, neutralizing antibody titers, and baseline factor VIII activity, has been conducted to assess the efficacy and safety of AAV vector gene therapy in hemophiliac patients. Nonetheless, there were some limitations. First, there was high heterogeneity that existed among the included studies. To mitigate the impact of this heterogeneity, we have used a random effects model throughout our analysis. Factors contributing to this heterogeneity included small sample sizes due to the rarity of hemophilia, patient population variability (such as age, race, and pre-existing conditions), differences in vector design (AAV serotypes, promoter selection, and transgene modifications), dosing strategies, and immunological factors (neutralizing antibodies, cytotoxic T-cell responses, and corticosteroid prophylaxis). Second, the included studies were all non-controlled trials with a small sample size, potentially introducing significant bias to our results, and thus, we only evaluated the efficacy and risk without definite conclusions. Third, hemophilia gene therapy studies often use single-arm trials due to the rare nature of the disease and ethical considerations, which restrict robust comparisons with standard treatments. This design can introduce selection bias and confounding factors, making it challenging to definitively attribute observed outcomes solely to the gene therapy. Future research should prioritize conducting more randomized controlled trials (RCTs) to rigorously assess the efficacy and safety of gene therapy compared to other treatments, such as emicizumab or extended half-life factor concentrates. Furthermore, the follow-up period is insufficient to assess the safety and long-term effectiveness of gene therapy, particularly in terms of the durability of factor activity levels and potential genotoxicity risks. Clotting factor levels may decline over time, highlighting the need for longer-term data to fully understand the therapeutic effectiveness and potential adverse effects.

Despite the above-mentioned limitations, our evidence-based analysis supports earlier studies demonstrating the advantages of AAV-based gene therapy in the treatment of hemophilia patients. Thus, additional trials are needed to demonstrate the efficacy and safety of gene therapy in all patients. Our current study provides only a reference to clinical practice and the development of more gene therapy drugs. We look forward to the development and application of more effective AAV-based gene therapy medications.

The field of gene therapy is witnessing a surge in the development of novel therapeutic products. Second-generation gene therapies represent a significant advancement, characterized by enhanced safety and efficacy profiles achieved through optimized delivery mechanisms, reduced immunogenicity, and refined transgene regulation. These innovations are not only improving the therapeutic outcomes for existing indications but also expanding the scope of gene therapy into diverse disease areas, including ophthalmology, diabetes, oncology, and beyond. The growing availability of these advanced gene therapy products holds the potential to transform the treatment landscape, offering a greater number of patients the prospect of long-term disease management and, in some cases, a cure.

## 5 Conclusion

In summary, our meta-analysis demonstrates the efficacy and safety of AAV-based gene therapy in hemophilia patients, providing evidence for its future clinical application.

However, since there are limited clinical data, future large-scale and multiple-center RCTs are required to confirm this conclusion.

## Data Availability

The original contributions presented in the study are included in the article/Supplementary material, further inquiries can be directed to the corresponding author.
