# Supplementary material for: Adeno-associated virus gene therapy for hemophilia: an update meta-analysis and systematic review
Source: Front Med (Lausanne). 2025 May 20;12:1580264. doi: 10.3389/fmed.2025.1580264 (PMC12130019; doi:10.3389/fmed.2025.1580264)
Supplement: Supplementary file 1 [file Data_Sheet_1.DOCX]

**Supplementary 2**


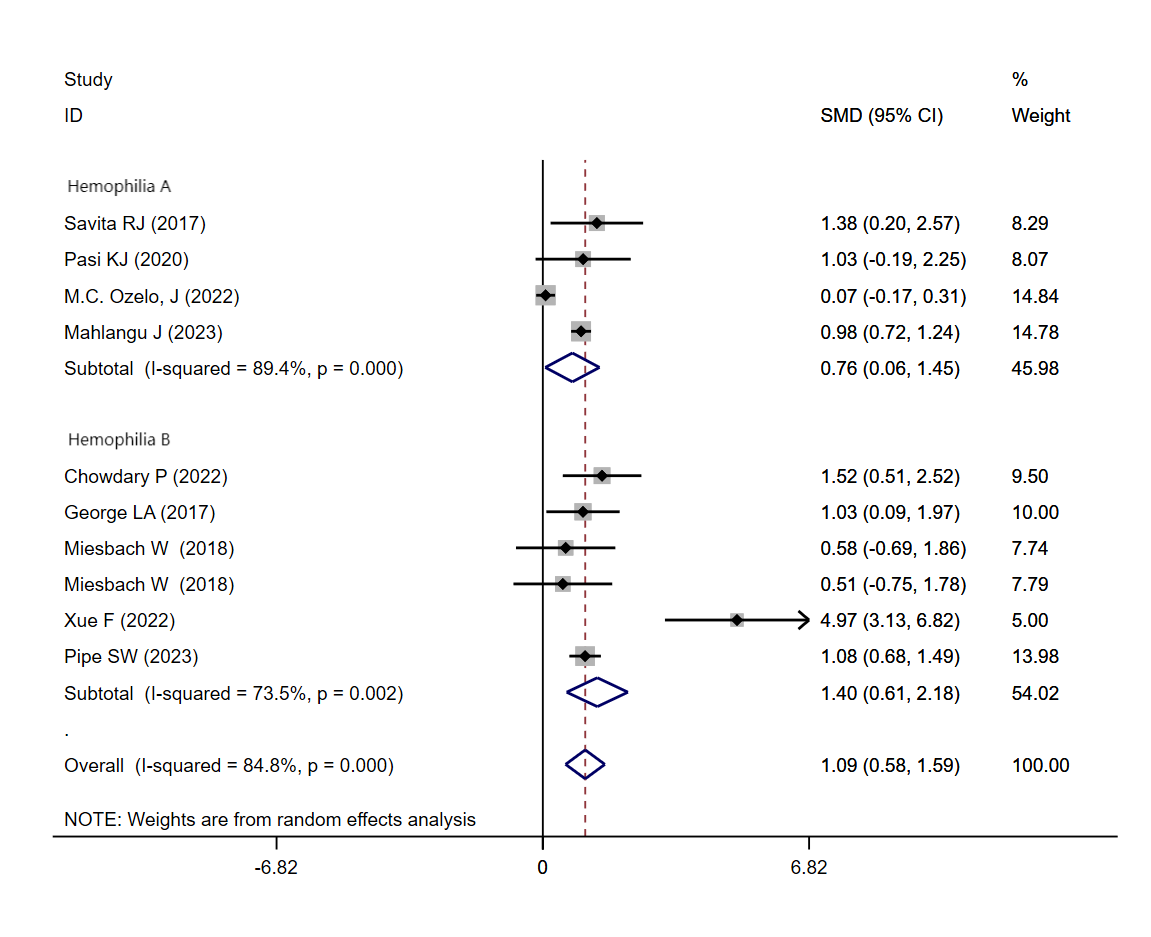


Supplementary Figure 2B


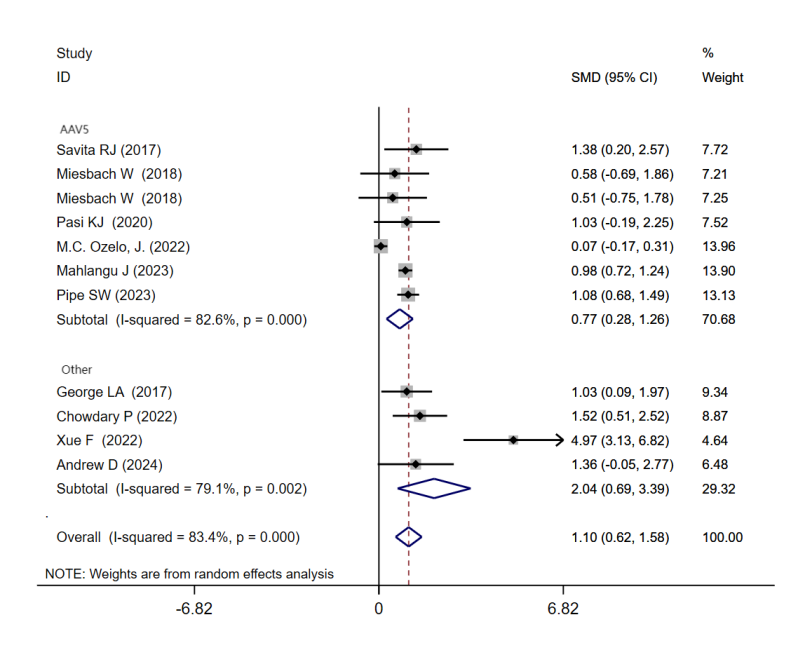


Supplementary Figure 2C


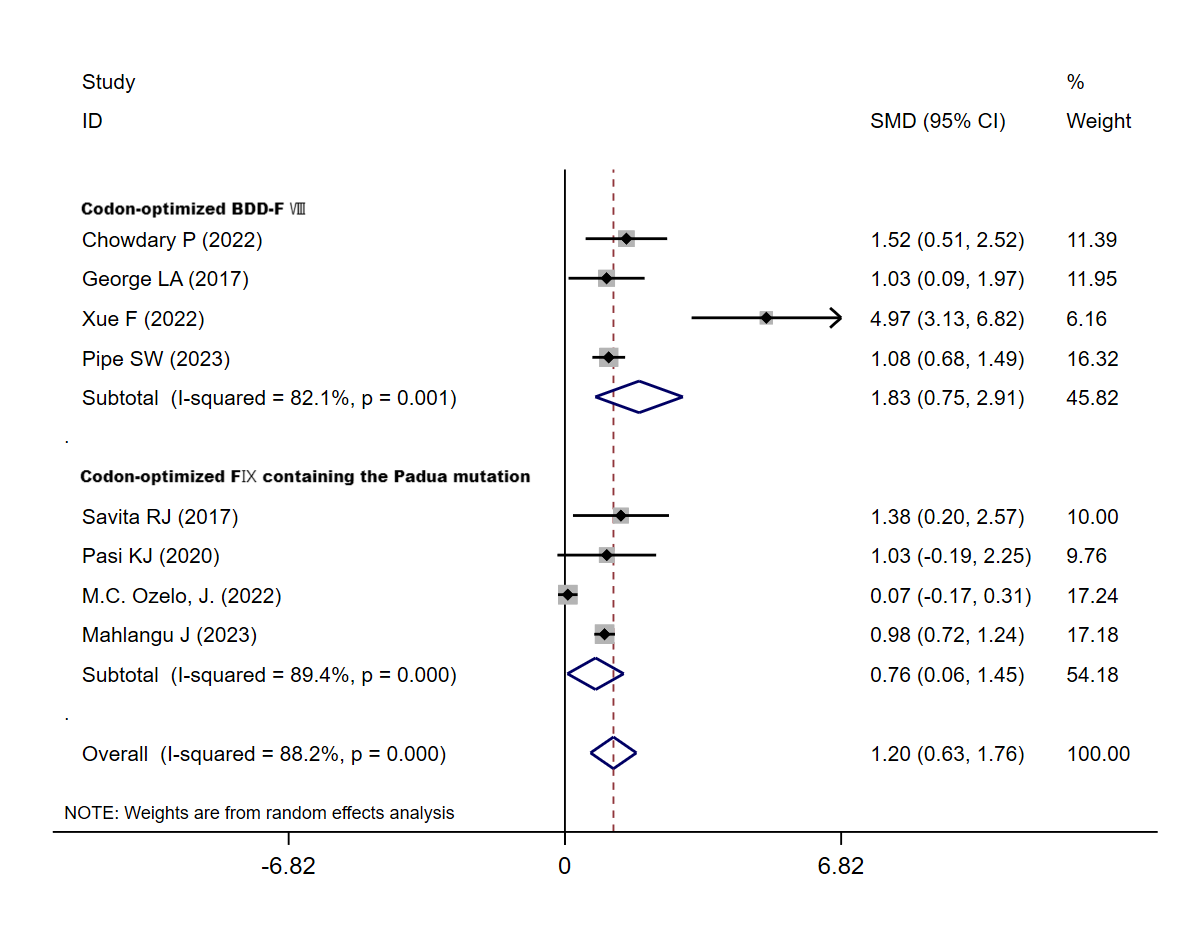


Supplementary Figure 2D


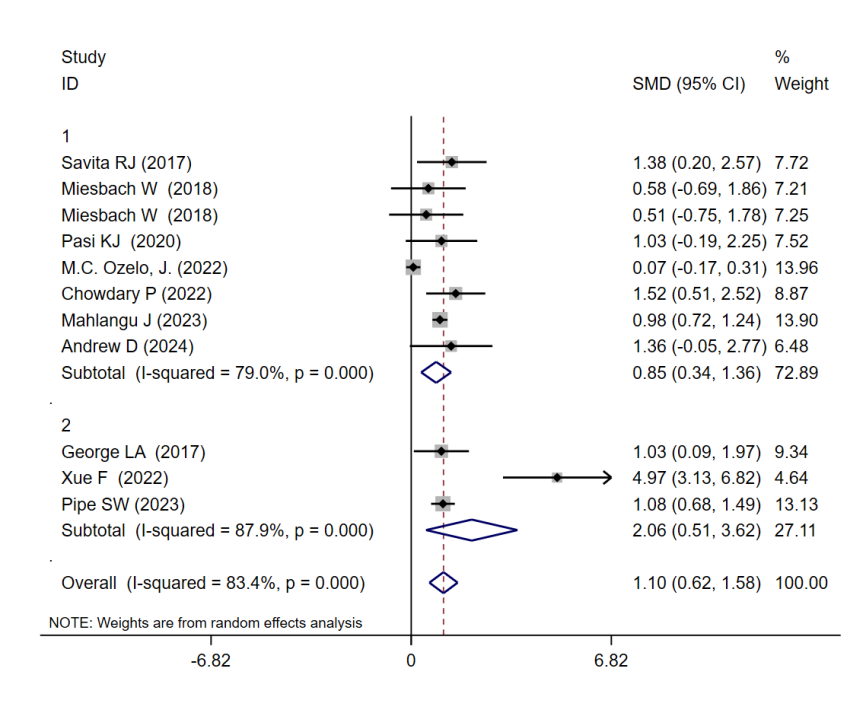


Supplementary Figure 2E


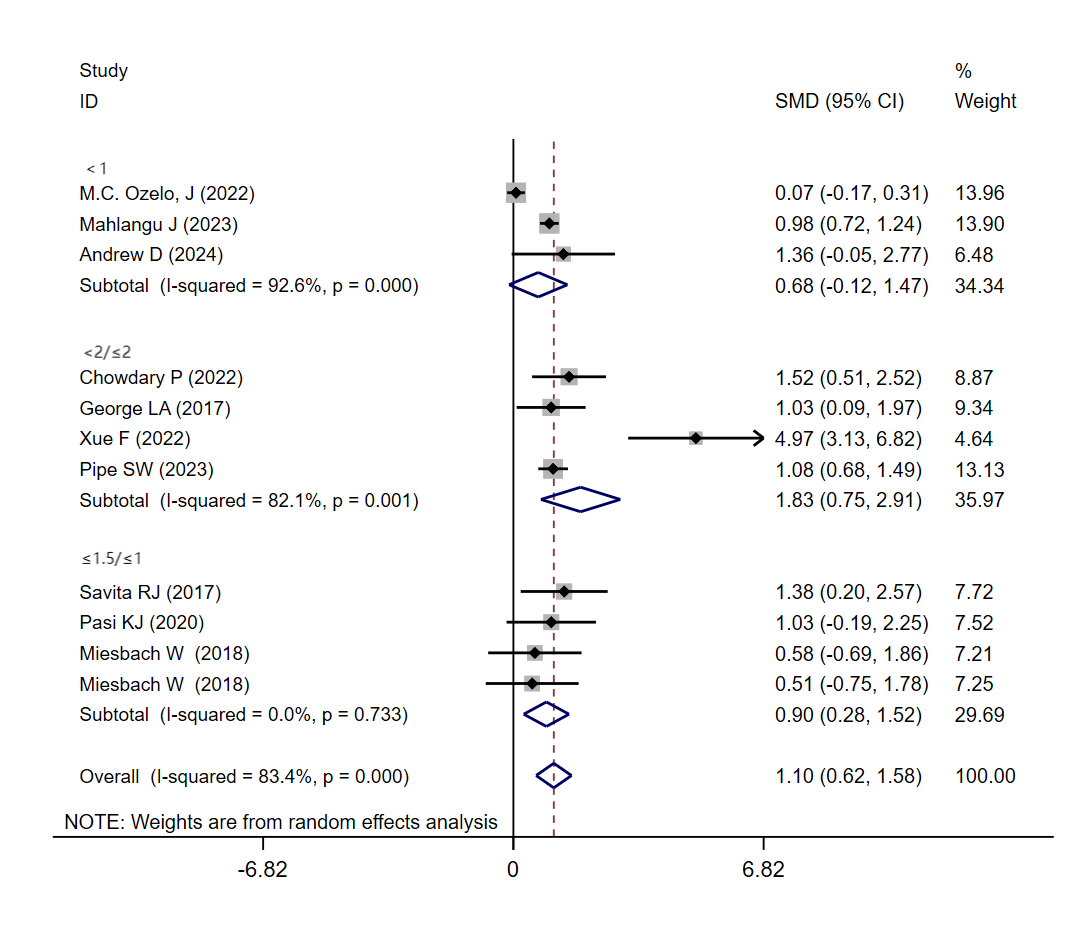


Supplementary Figure 2F

**Supplementary 3**


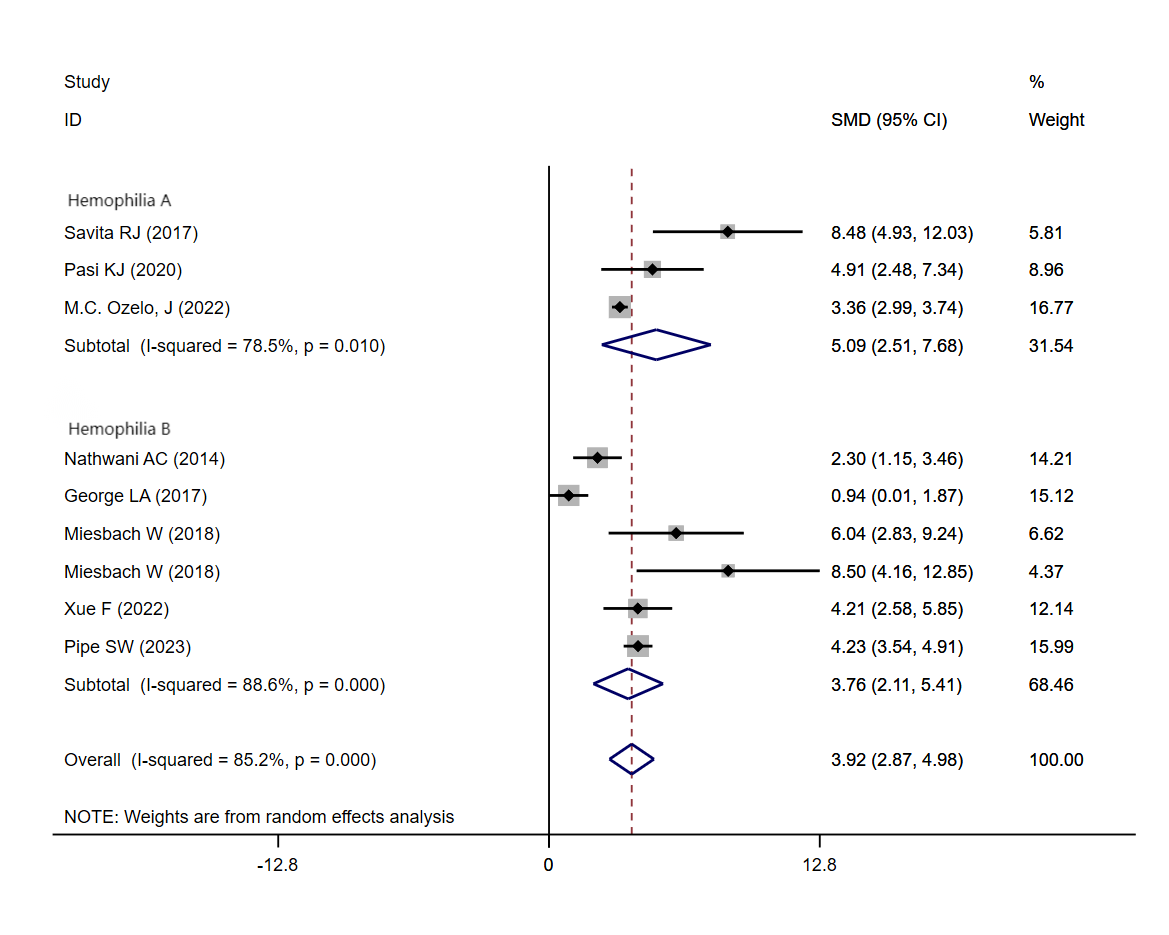


Supplementary Figure 3B


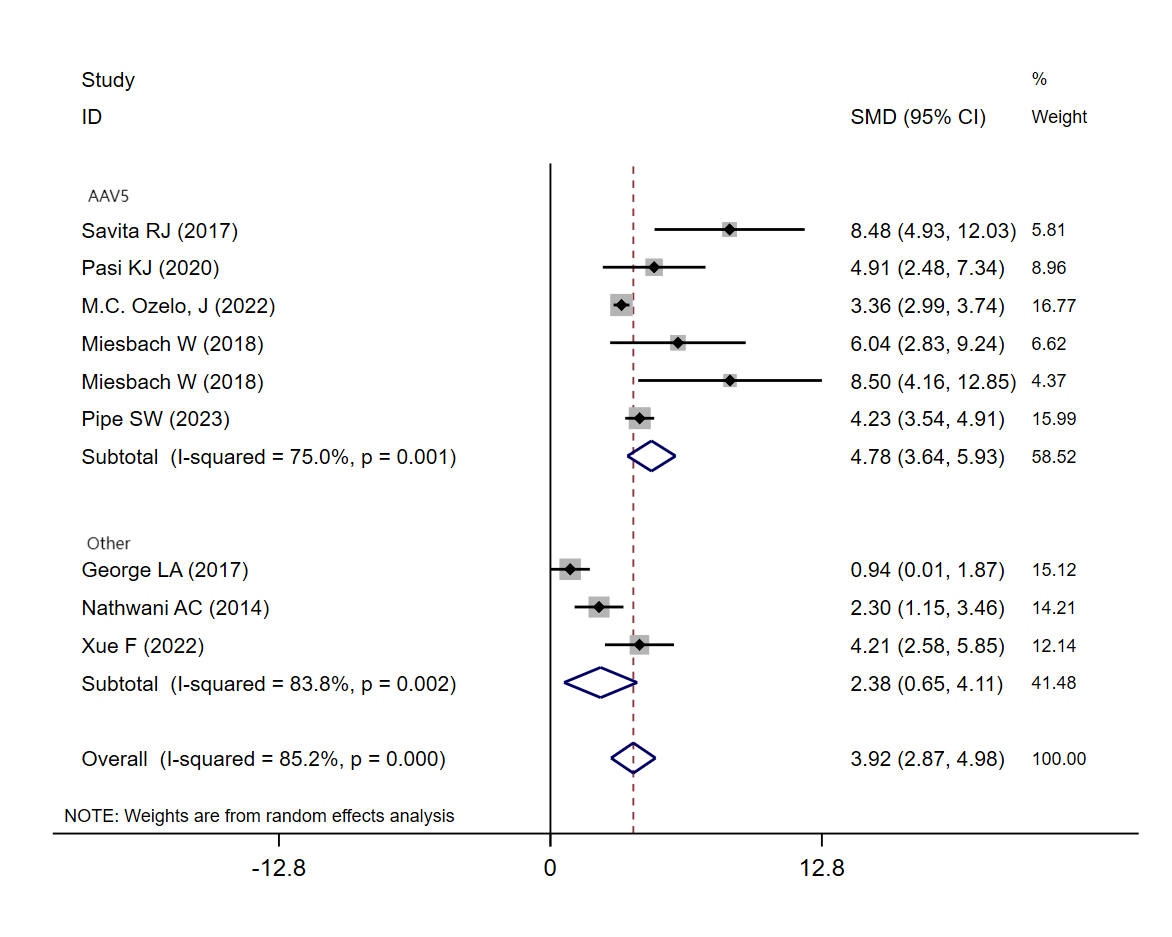


Supplementary Figure 3C


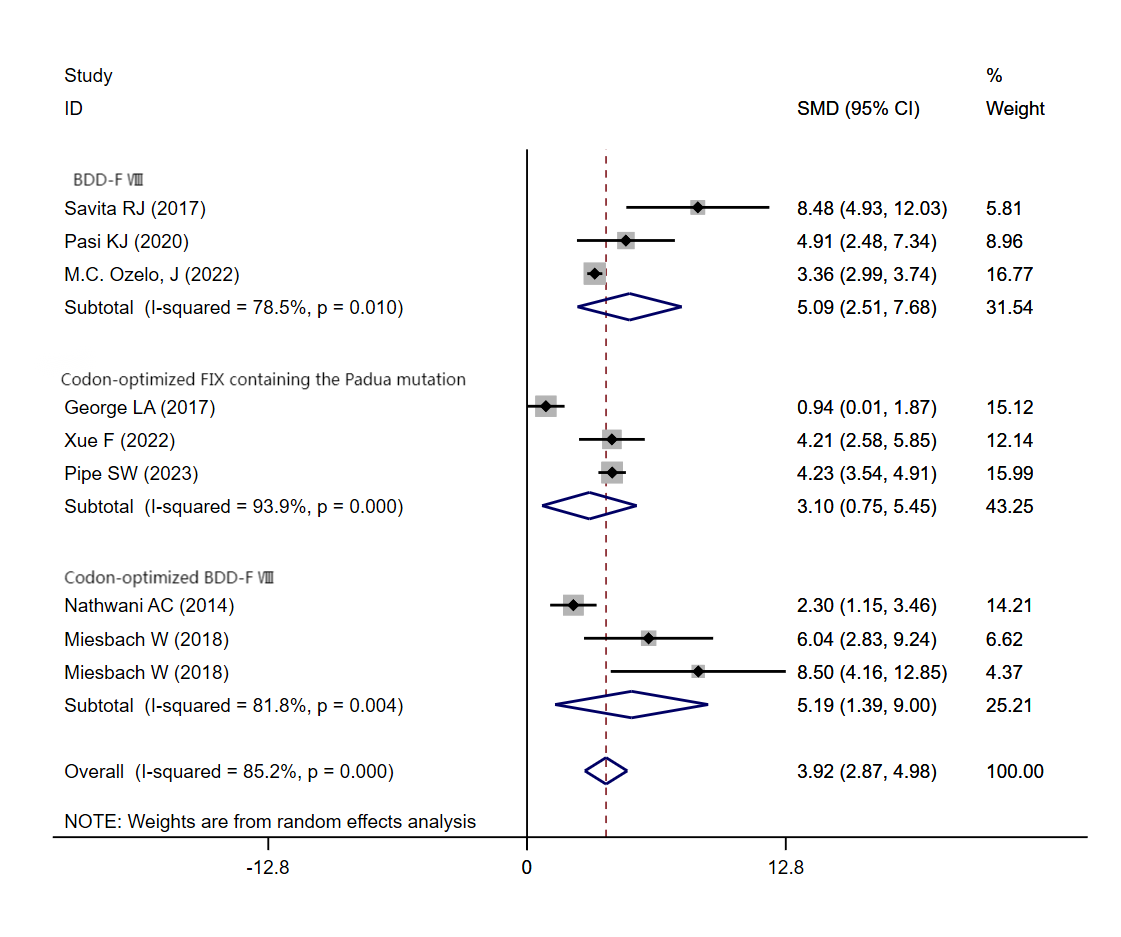


Supplementary Figure 3D


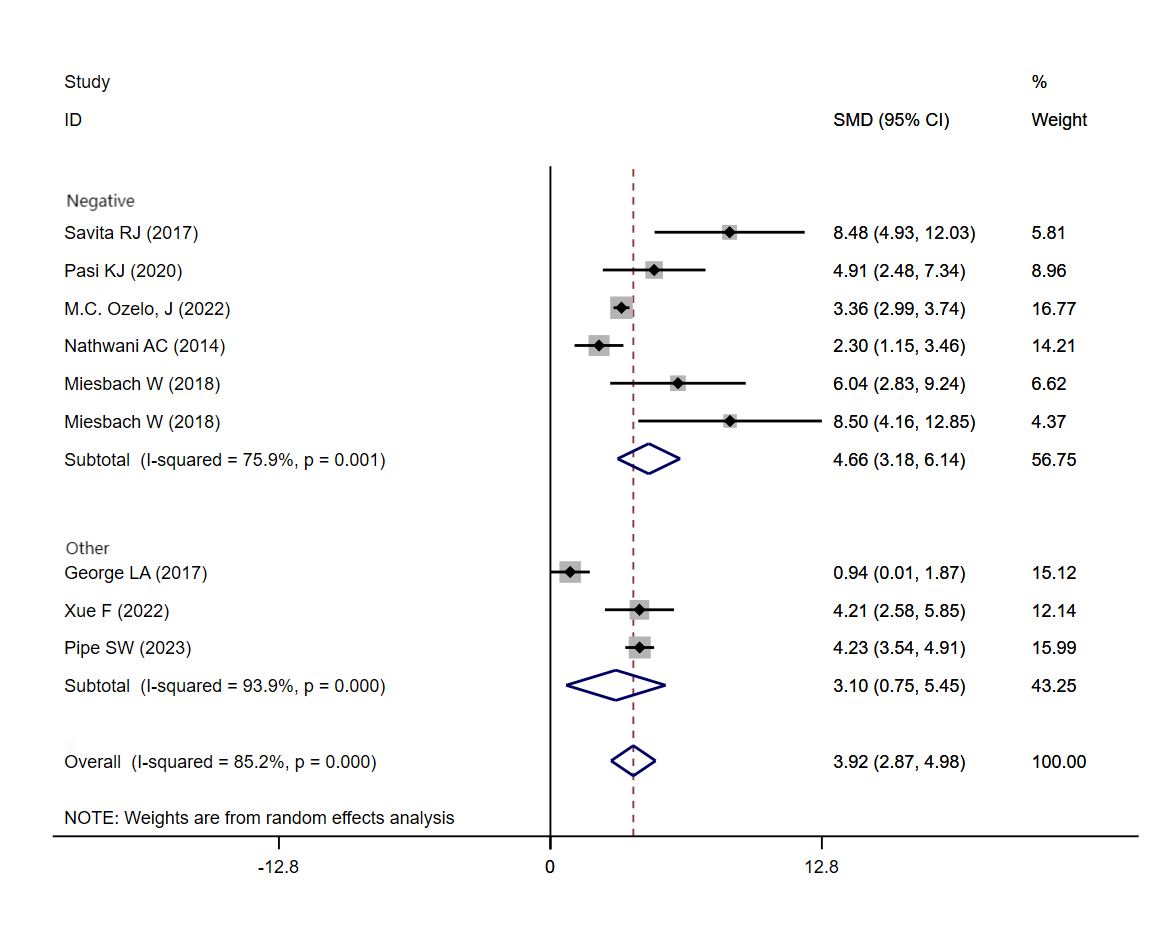


Supplementary Figure 3E


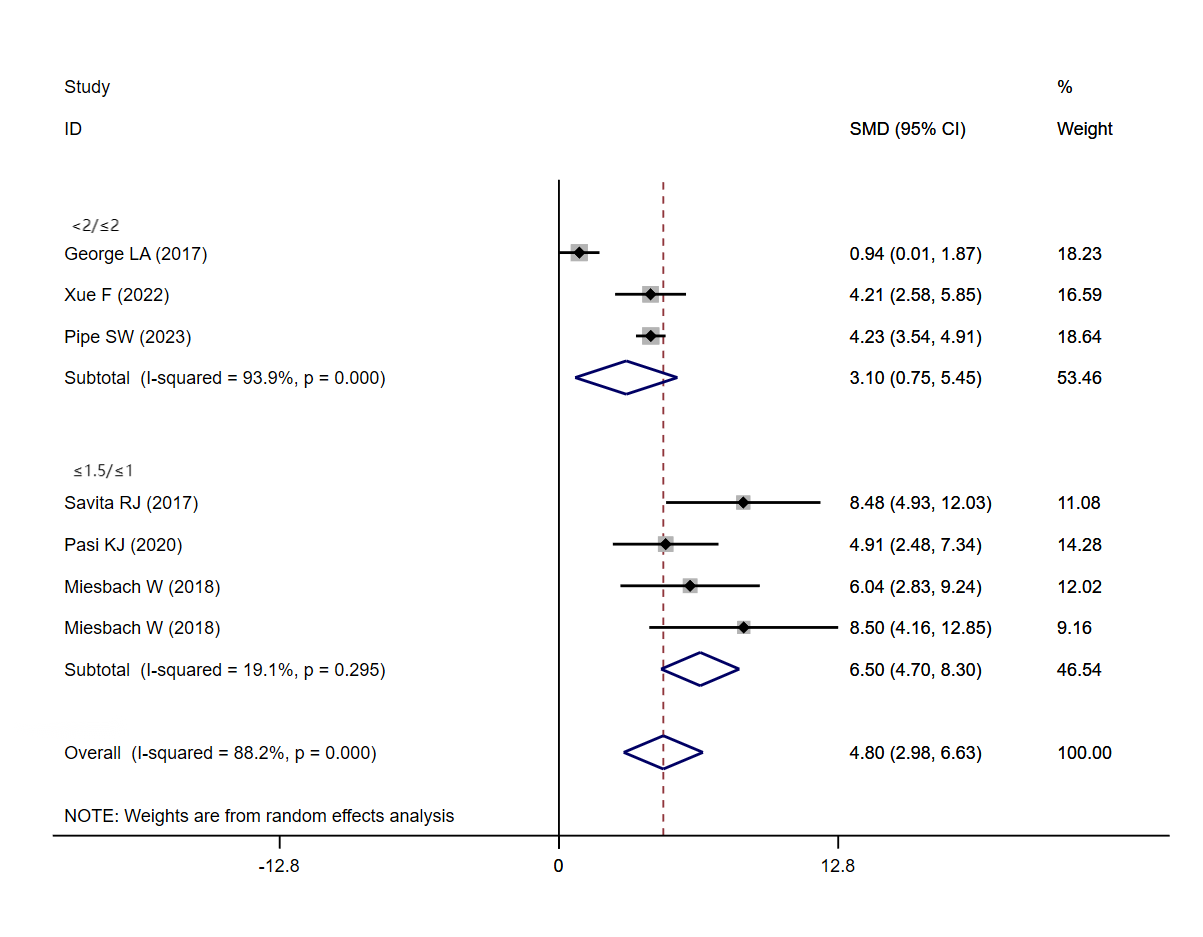


Supplementary Figure 3F

**Supplementary 4**


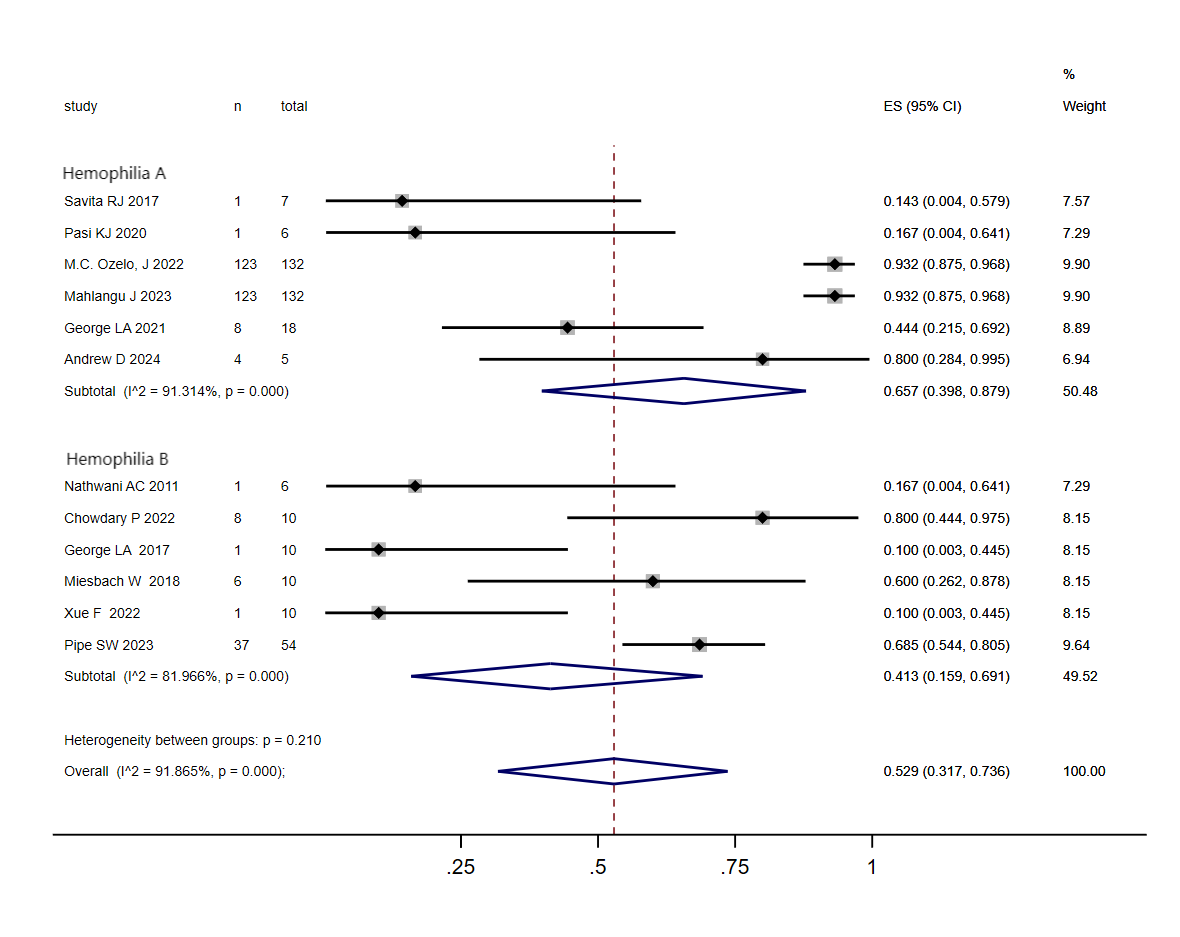


Supplementary Figure 4B


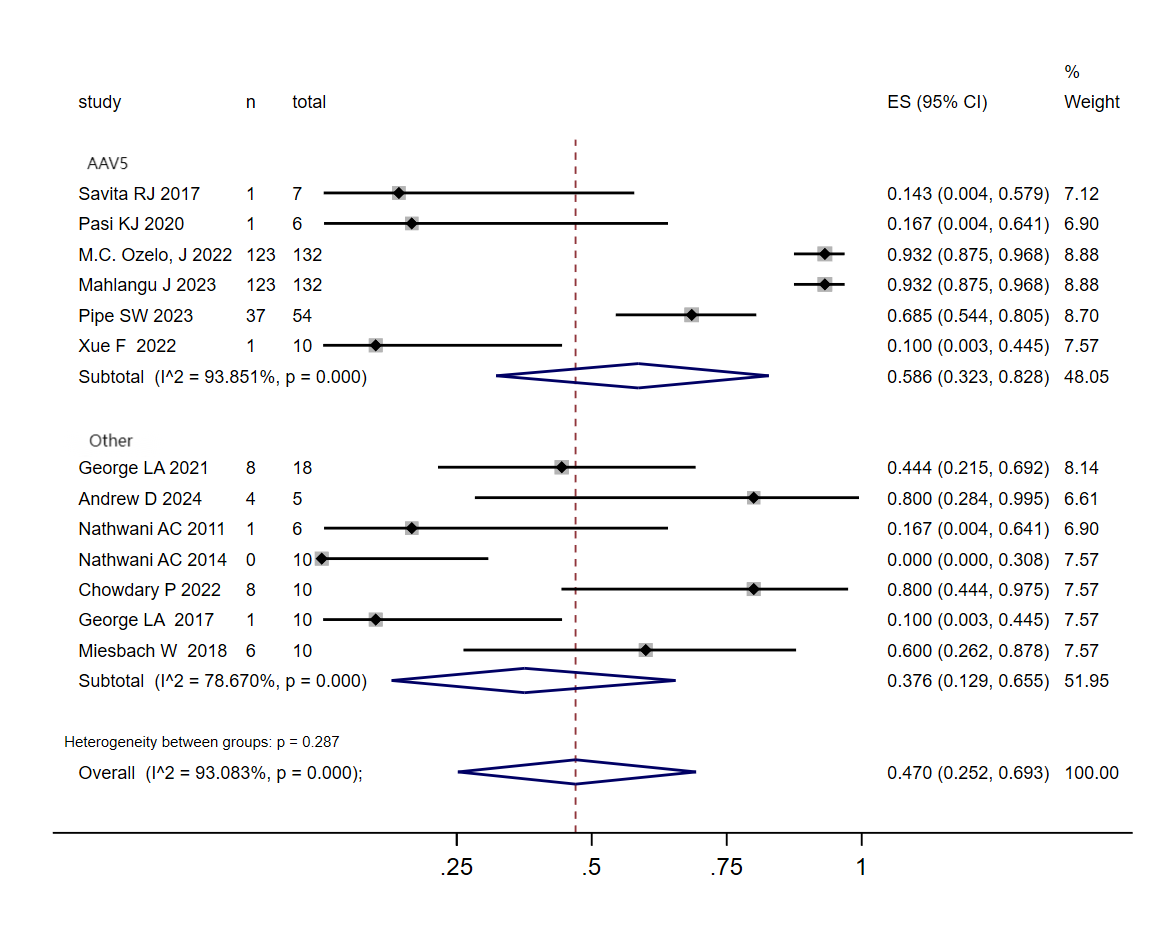


Supplementary Figure 4C


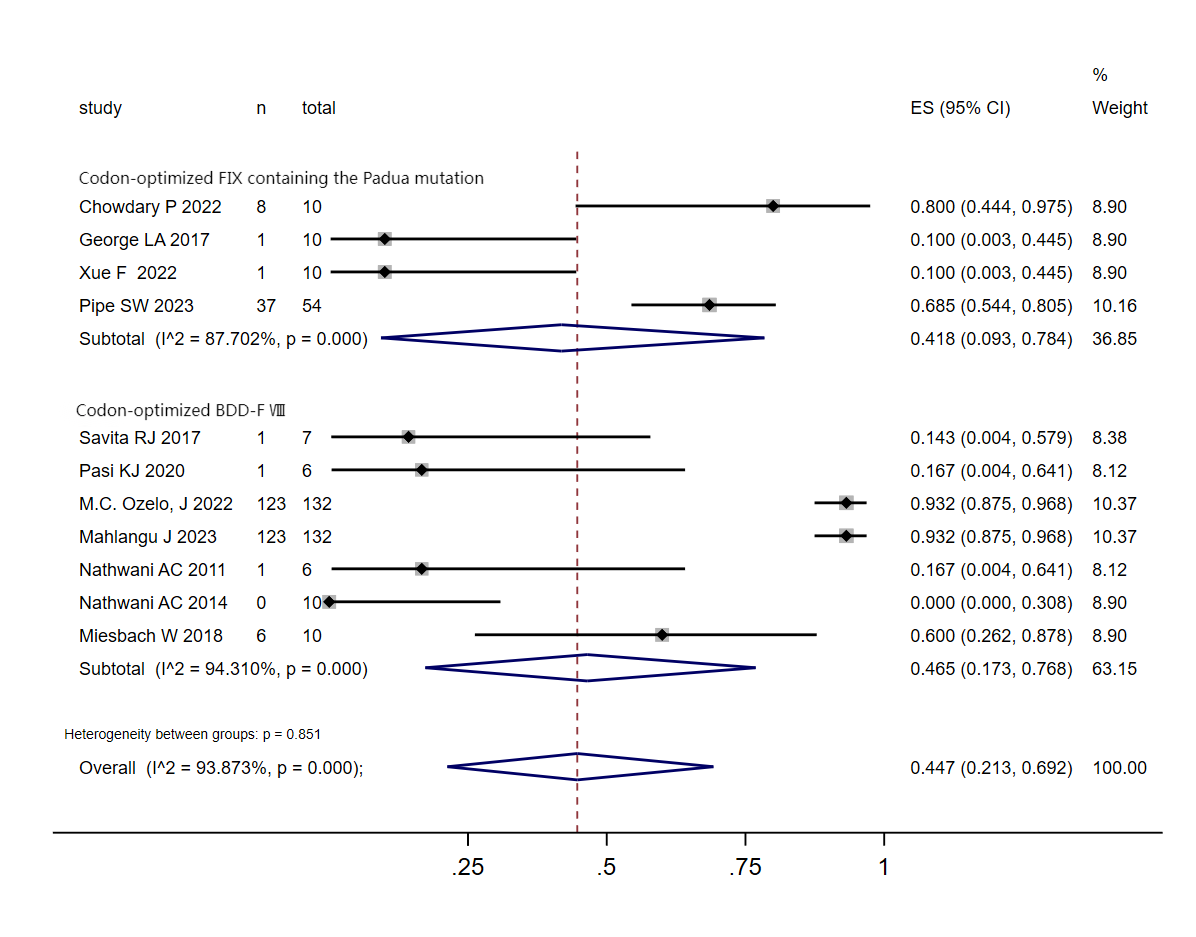


Supplementary Figure 4D


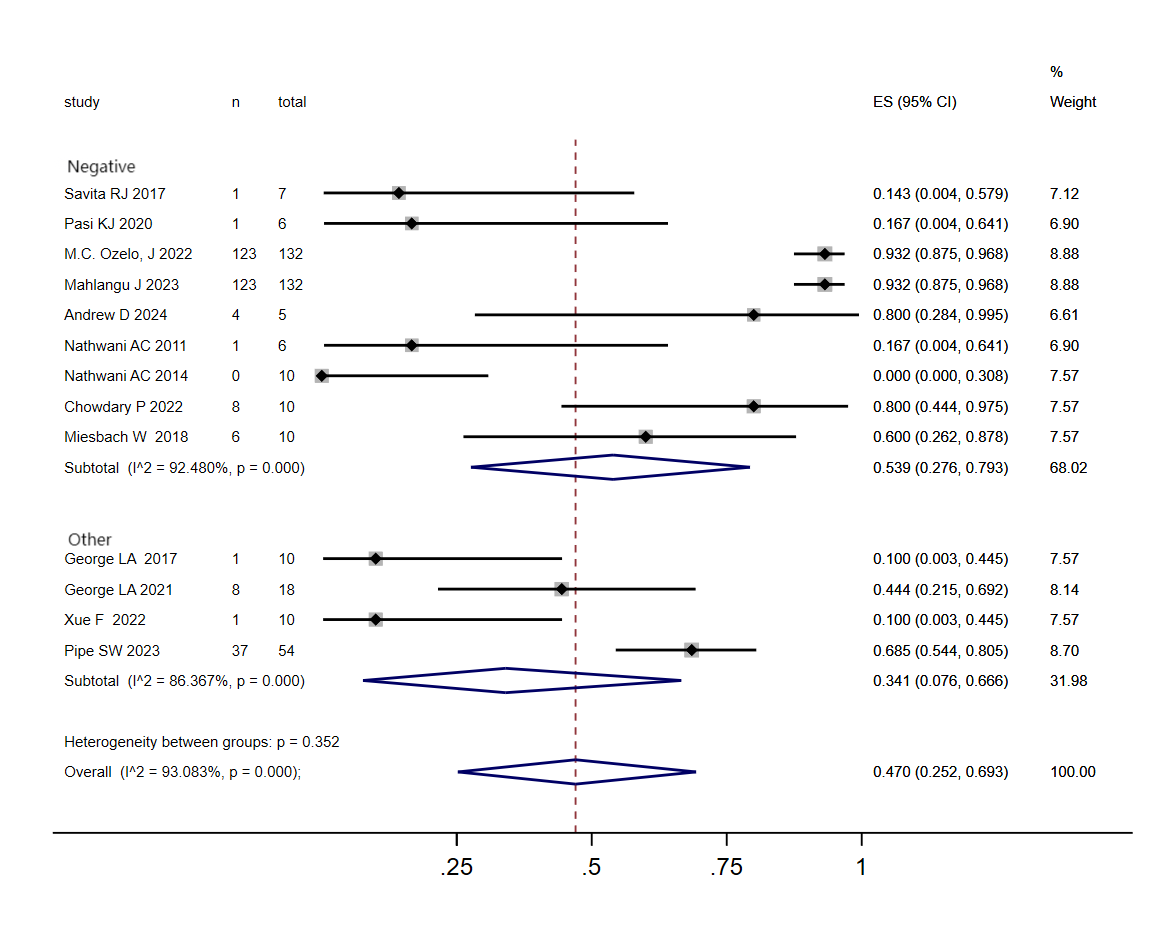


Supplementary Figure 4E


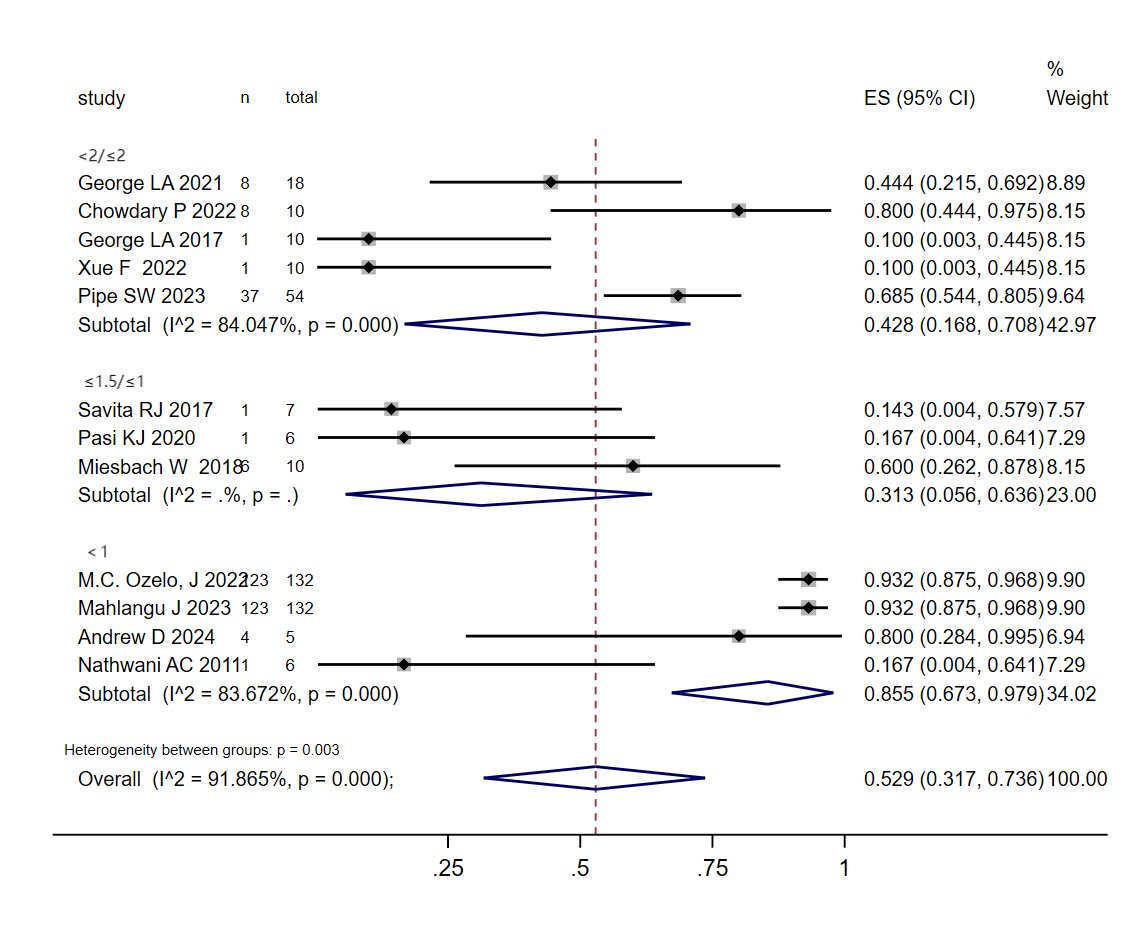


Supplementary Figure 4F

**Supplementary 5**


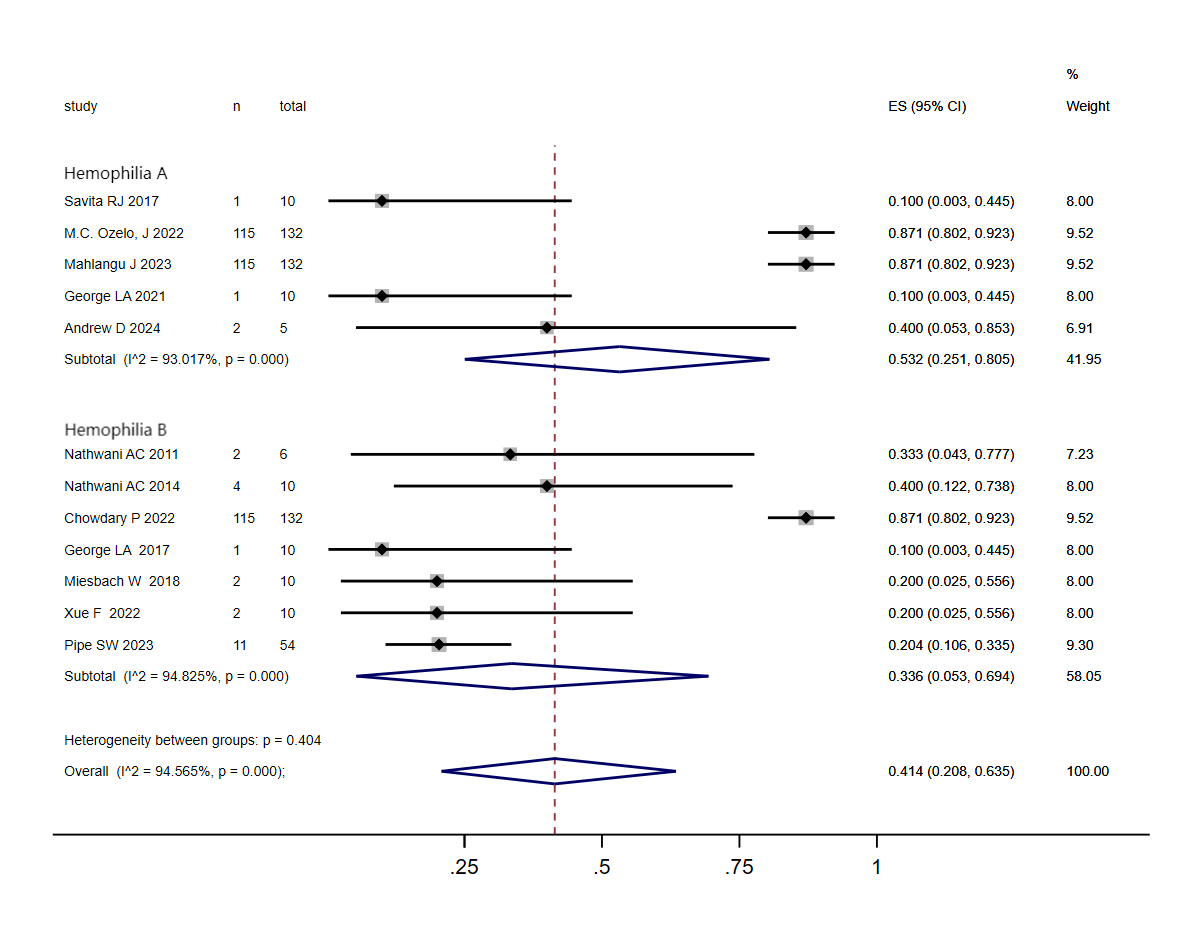


Supplementary Figure 5A


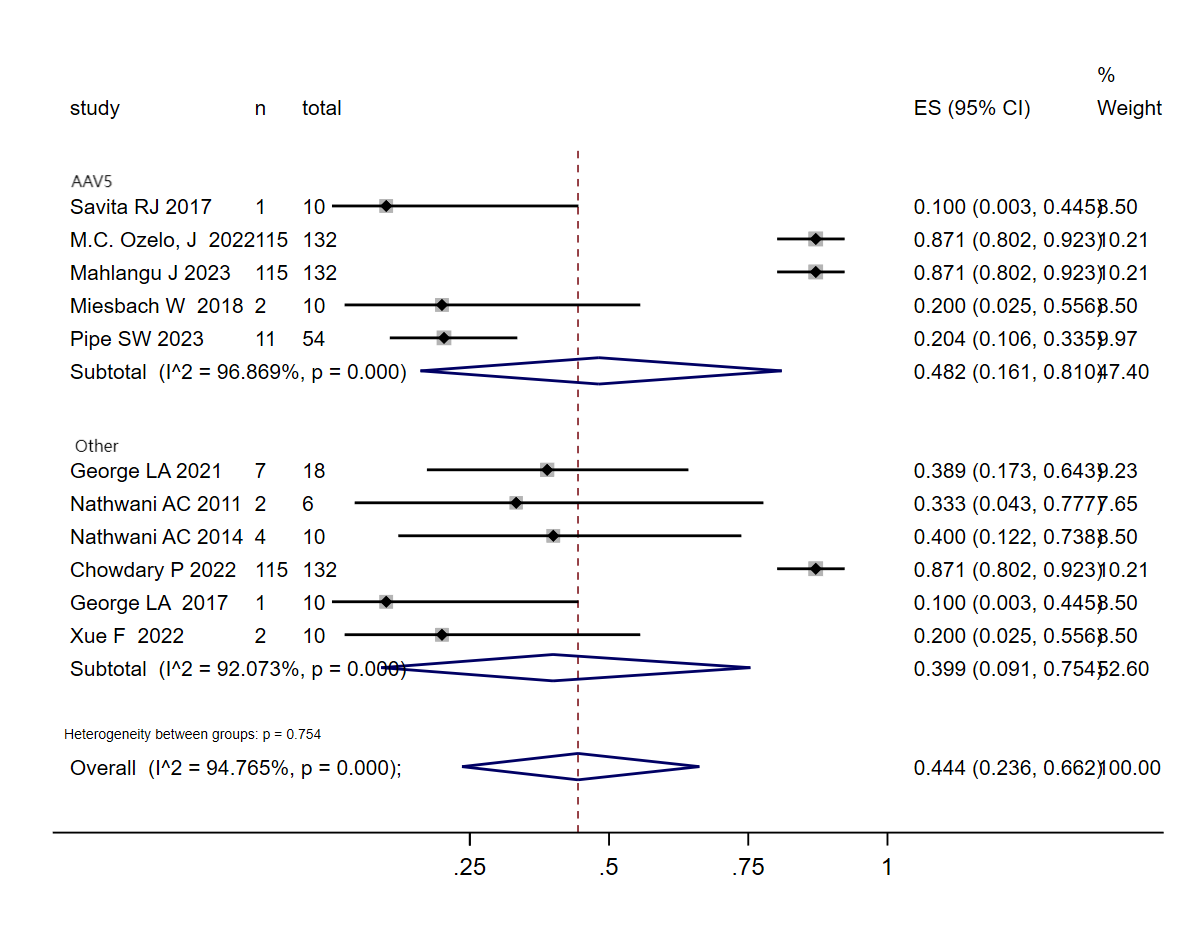


Supplementary Figure 5B


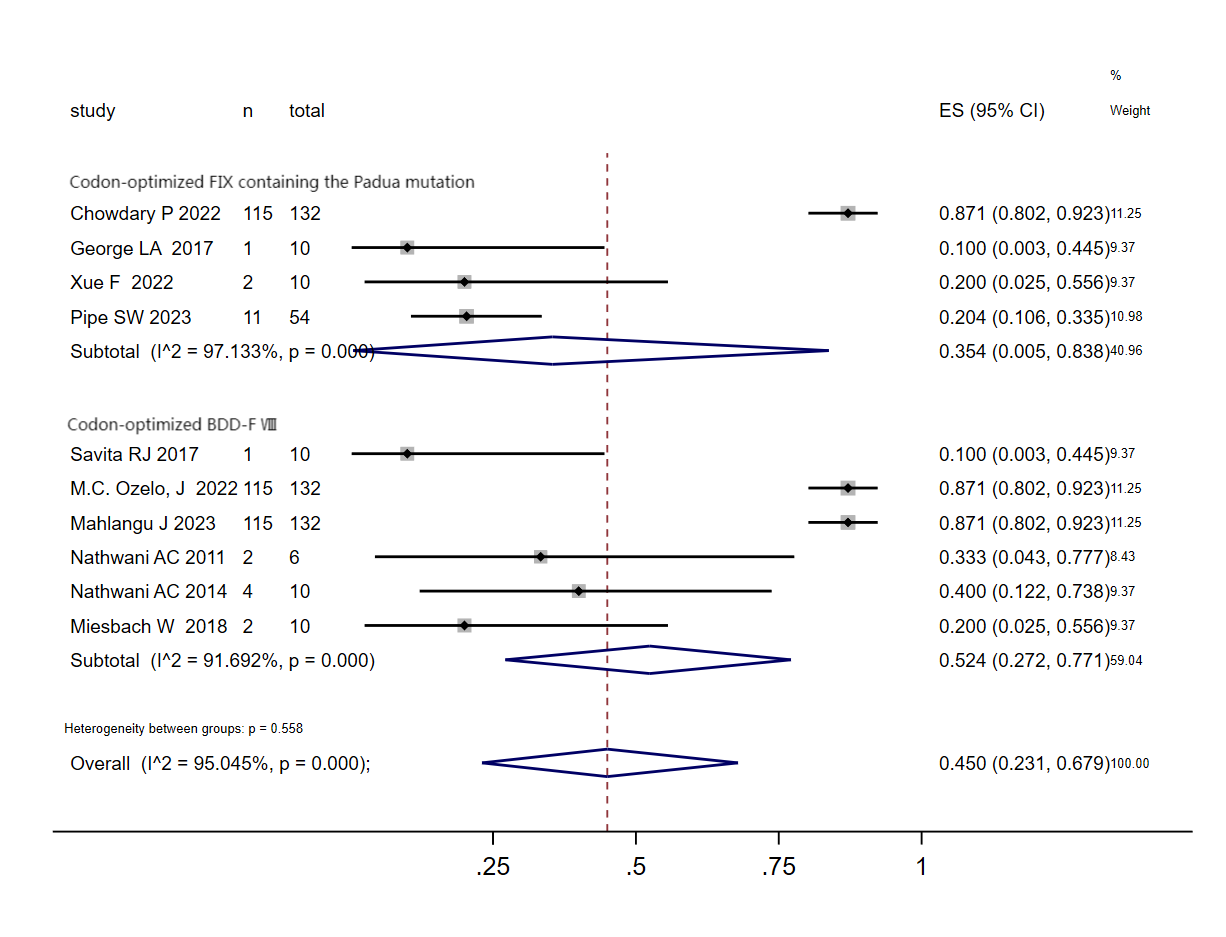


Supplementary Figure 5C


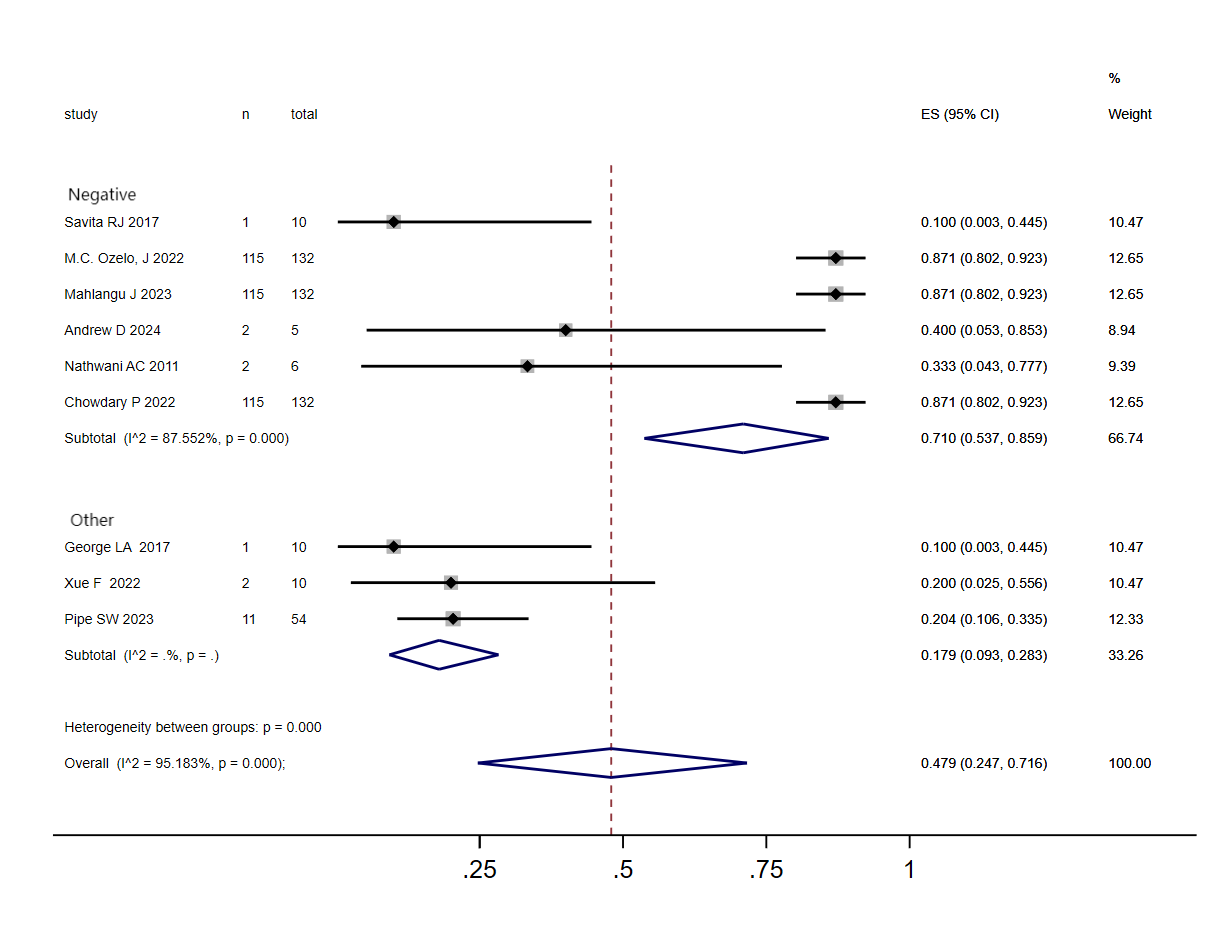


Supplementary Figure 5D


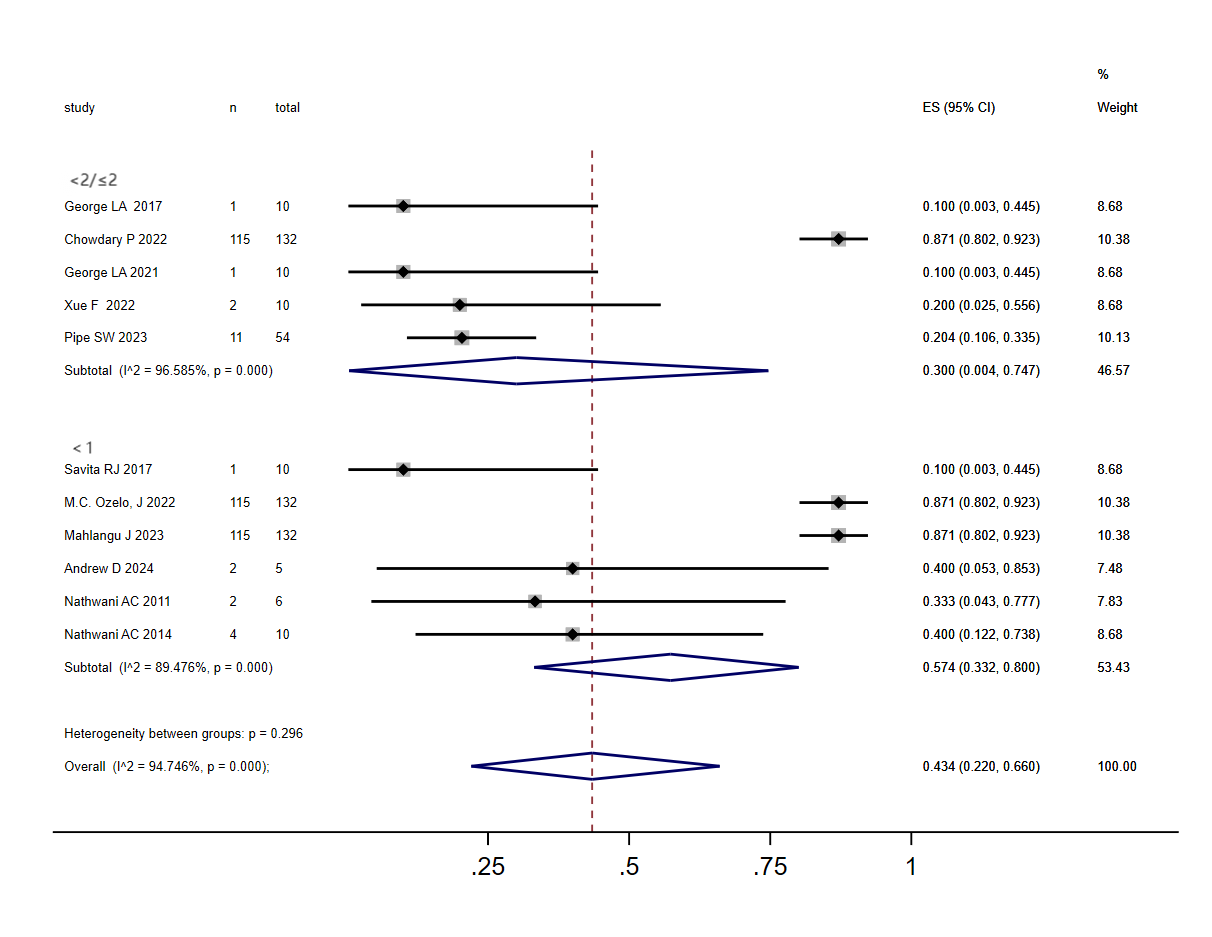


Supplementary Figure 5E

**Supplementary 6**


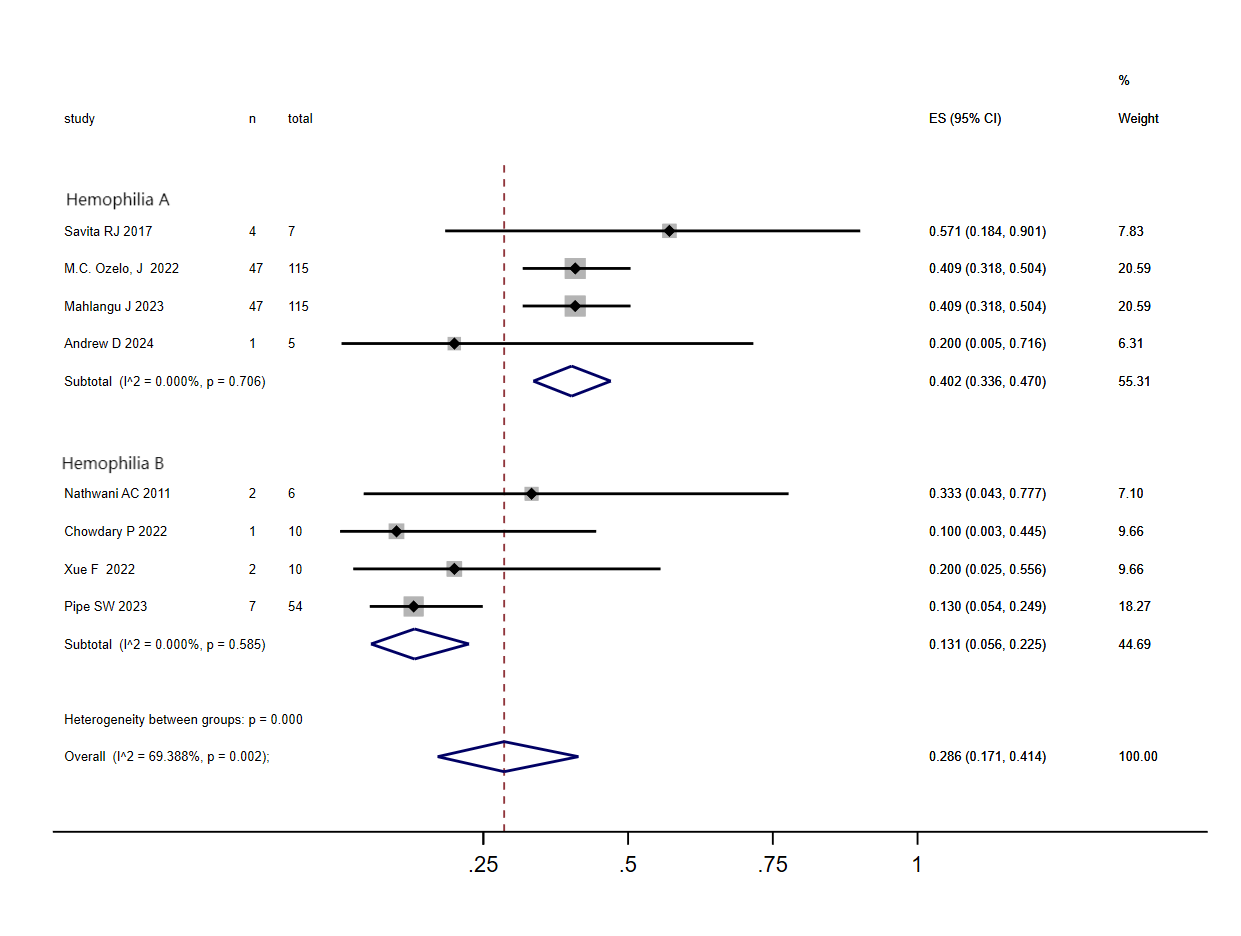


Supplementary Figure 6A


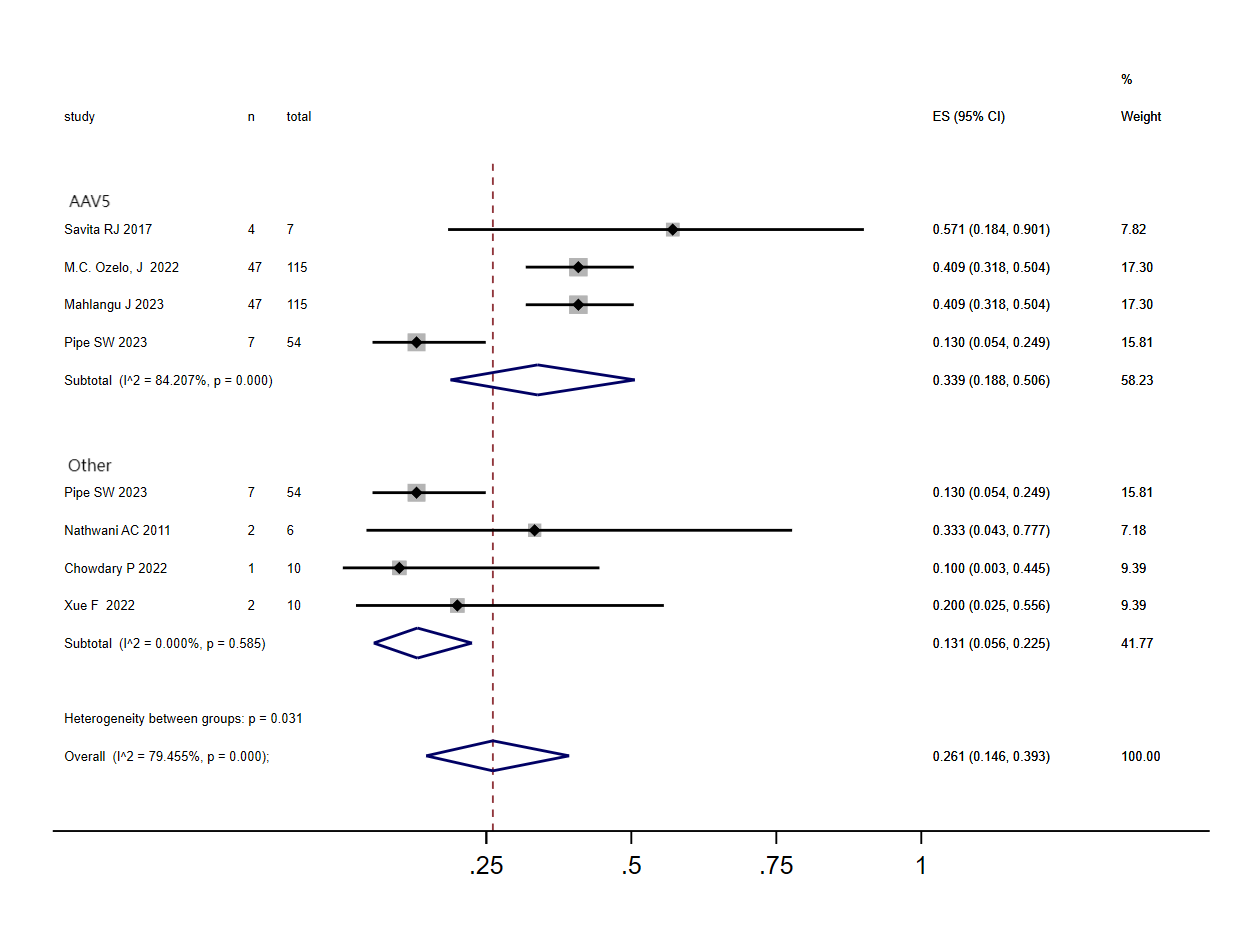


Supplementary Figure 6B


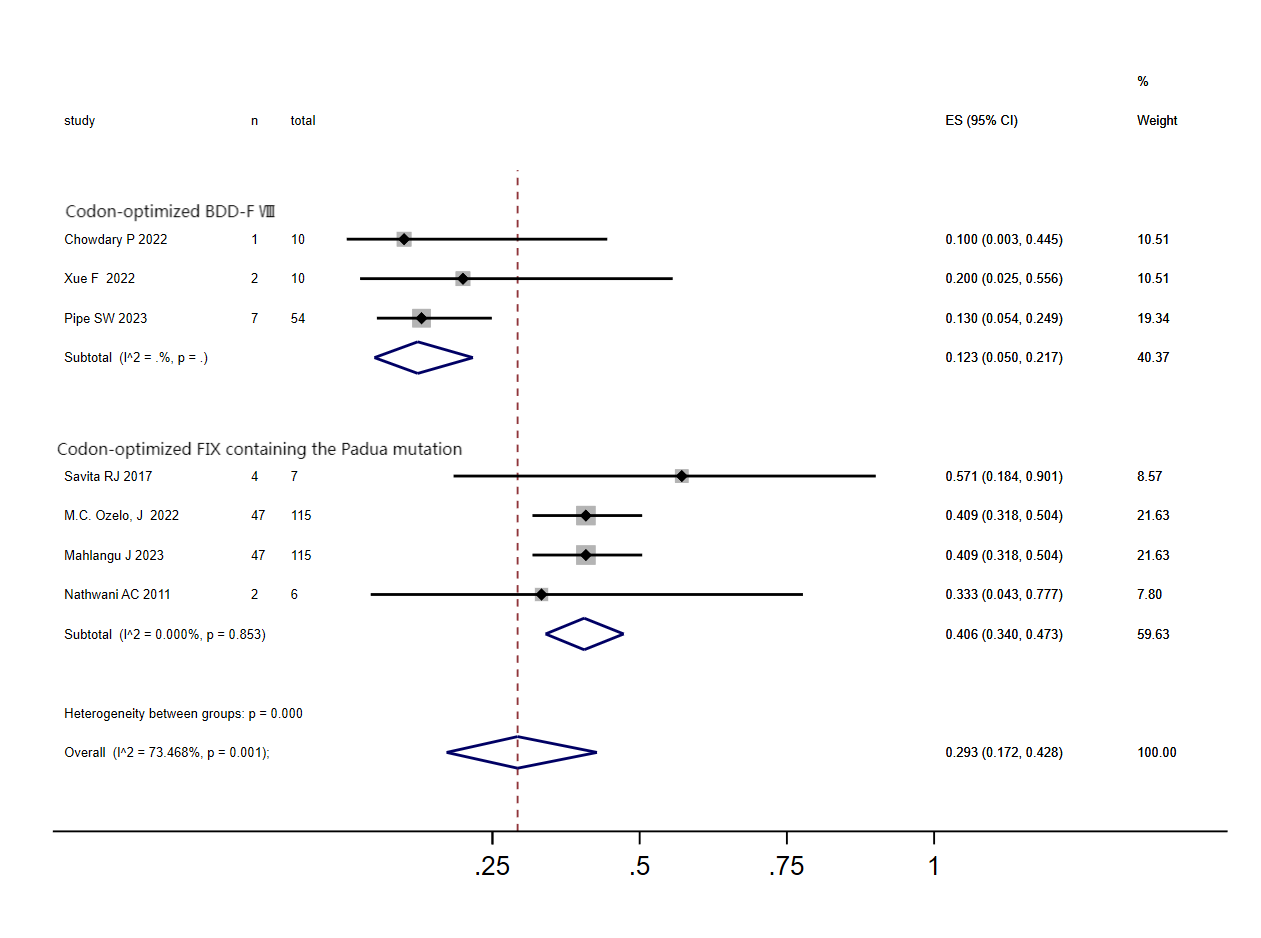


Supplementary Figure 6C


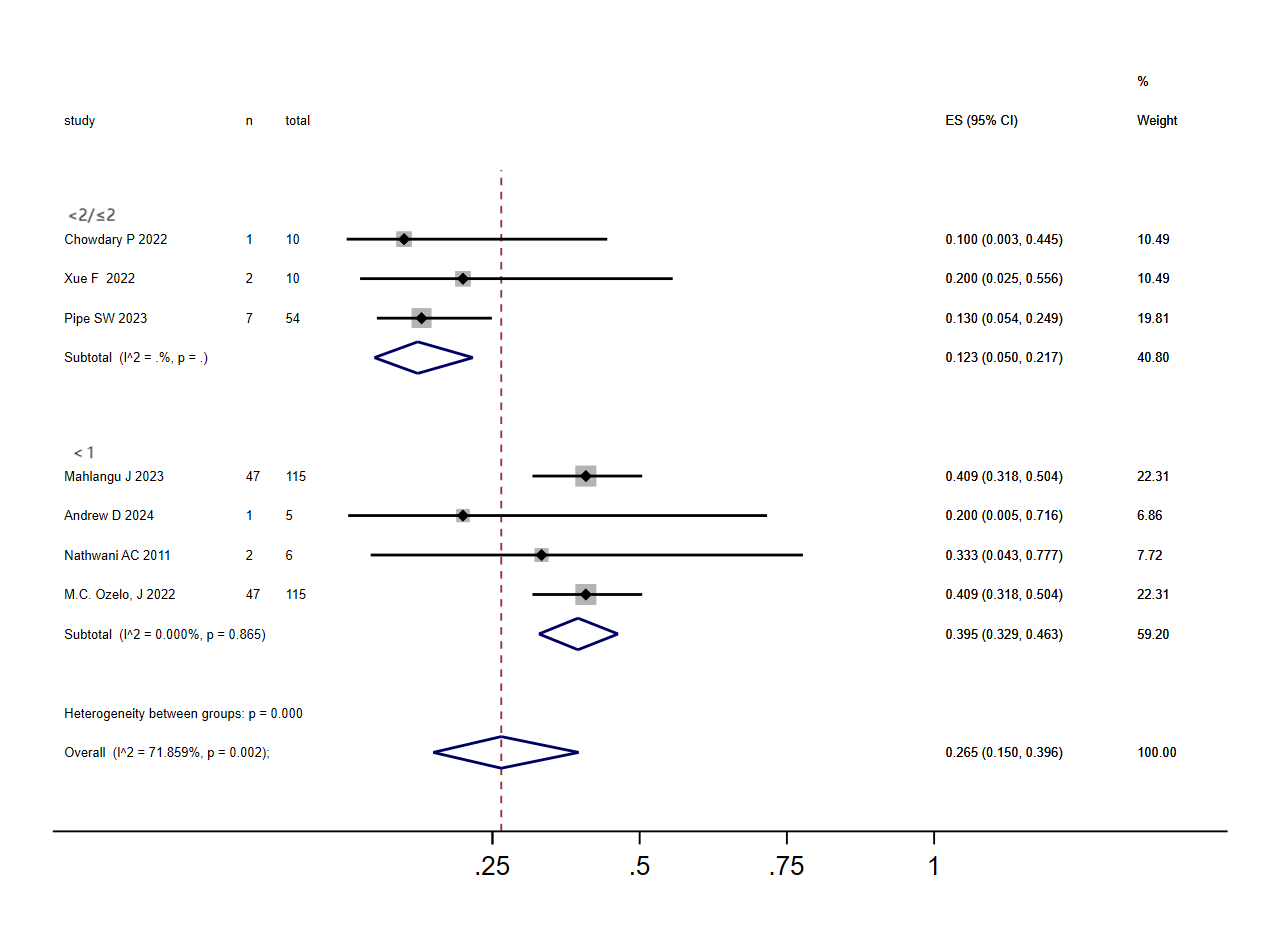


Supplementary Figure 6D

**Supplementary 7**

| Study | Year | MINORS score |
| --- | --- | --- |
| Savita RJ | 2017 | 13 |
| Pasi KJ | 2020 | 14 |
| M.C. Ozelo, J | 2022 | 13 |
| Mahlangu J | 2023 | 14 |
| George LA | 2021 | 14 |
| Andrew D | 2024 | 12 |
| Nathwani AC | 2011 | 13 |
| Nathwani AC | 2014 | 14 |
| Chowdary P | 2022 | 14 |
| George LA | 2017 | 14 |
| Miesbach W | 2018 | 12 |
| Xue F | 2022 | 14 |
| Pipe SW | 2023 | 14 |
